# Ophiostomatoid fungi associated with pine bark beetles and infested pines in south-eastern Australia, including *Graphilbum ipis-grandicollis* sp. nov.

**DOI:** 10.1186/s43008-021-00076-w

**Published:** 2021-09-01

**Authors:** Conrad Trollip, Angus J. Carnegie, Quang Dinh, Jatinder Kaur, David Smith, Ross Mann, Brendan Rodoni, Jacqueline Edwards

**Affiliations:** 1 School of Applied Systems Biology, La Trobe University, Bundoora, VIC 3083 Australia; 2 Department of Jobs, Precincts and Regions, Agriculture Victoria Research, AgriBio Centre, Bundoora, VIC 3083 Australia; 3 Forest Science, NSW Department of Primary Industries – Forestry, Parramatta, NSW 2150 Australia; 4 Department of Jobs, Precincts and Regions, Biosecurity and Agricultural Services, Agriculture Victoria, Cranbourne, VIC 3977 Australia

**Keywords:** *Ceratocystiopsis*, *Graphilbum*, *Leptographium*, *Ophiostoma*, *Raffaelea*, *Sporothrix*, *Graphium*, One new taxon

## Abstract

The ophiostomatoid fungi are an assemblage of ascomycetes which are arguably best-known for their associations with bark and ambrosia beetles (*Curculonidae*) and blue stain (sap stain) of many economically important tree species. These fungi are considered a significant threat to coniferous forests, which has resulted in numerous studies characterising the diversity of bark beetles and their ophiostomatoid associates globally. The diversity of ophiostomatoid fungi present in Australian pine plantations, however, remains largely undetermined. The aims of this study were therefore to reconsider the diversity of ophiostomatoid fungi associated with *Pinus* in Australia, and to establish the baseline of expected taxa found within these plantation ecosystems. To achieve this, we reviewed Australian plant pathogen reference collections, and analysed samples collected during forest health surveillance programs from the major pine growing regions in south-eastern Australia. In total, 135 ophiostomatoid isolates (15 from reference collections and 120 collected during the current study) were assessed using morphological identification and ITS screening which putatively distinguished 15 taxonomic groups. Whole genome sequencing (WGS) of representative isolates from each taxon was performed to obtain high-quality sequence data for multi-locus phylogenetic analysis. Our results revealed a greater than expected diversity, expanding the status of ophiostomatoid fungi associated with *Pinus* in Australia to include 14 species from six genera in the *Ophiostomatales* and a single species residing in the *Microascales*. While most of these were already known to science, our study includes seven first records for Australia and the description of one new species, *Graphilbum ipis-grandicollis* sp. nov*..* This study also provides an early example of whole genome sequencing (WGS) approaches replacing traditional PCR-based methods for taxonomic surveys. This not only allowed for robust multi-locus sequence extraction during taxonomic assessment, but also permitted the rapid establishment of a curated genomic database for ophiostomatoid fungi which will continue to aid in the development of improved diagnostic resources and capabilities for Australian biosecurity.

**Supplementary Information:**

The online version contains supplementary material available at 10.1186/s43008-021-00076-w.

## Introduction

Fungi within *Ophiostomatales* and *Microascales* are best known for their associations with arthropod vectors and include examples of some of the most devastating fungal-insect symbioses known to plant pathologists over the past century (Fisher et al. 2012; Wingfield et al. 2017b; Brasier and Webber 2019). Notable examples include the Dutch elm disease pathogens, *Ophiostoma ulmi* and *O. novo-ulmi* (Santini and Faccoli 2015; Brasier and Webber 2019), the laurel wilt pathogen *Raffaelea lauricola* (Harrington et al. 2008) as well as the numerous pathogens belonging to *Ceratocystis* which cause tree mortality in natural and agricultural ecosystems (Roux et al. 2007; Wingfield et al. 2017b; Tsopelas et al. 2017). In a recent review on novel associations for members of *Ophiostomatales* and *Microascales*, Wingfield et al. (2017b) highlight the numerous biological and anthropogenic factors that influence the dispersal of these fungi and their vectors globally; a major feature of the ever-increasing threat these fungi pose to global biosecurity.

Despite being formally recognised as two distinct orders in the *Sordariomycetes*, species belonging to *Ophiostomatales* and *Microascales* share a long and complicated taxonomic history and are collectively referred to as the ophiostomatoid fungi (Wingfield et al. 1993; Seifert et al. 2013). This is due to similarities shared across their biology, particularly in key morphological characters, that is believed to have been driven by convergent evolution in adaptation to insect-mediated dispersal (De Beer et al. 2013; Wingfield et al. 2017b). Ophiostomatoid fungi are commonly associated with bark (*Coleoptera*: *Scolytinae*) and ambrosia (*Curculonidae: Scolytinae, Platypodinae*) beetles (Kirisits 2004; Hofstetter et al. 2015), where a greater dependency and specificity is apparent for *Ophiostomatales* compared to *Microascales* (Wingfield et al. 2017b). Ophiostomatoid genera that are most commonly associated with beetles include: *Ambrosiella, Endoconidiophora* and *Graphium* in *Microascales*; and *Affroraffaelea, Aureovirgo*, *Ceratocystiopsis, Fragosphaeria, Graphilbum, Leptographium*, *Ophiostoma*, *Raffaelea,* and *Sporothrix* of *Ophiostomatales* (Hyde et al. 2020).

While not all ophiostomatoid fungi are responsible for tree mortality, many are well recognized as the causal agents of blue stain (sap stain) in the wood of economically important tree hosts (Kirisits 2004; Seifert et al. 2013). This is particularly true for pine (*Pinus*) plantations globally (Seifert et al. 2013; de Errasti et al. 2018; Jankowiak et al. 2021). Systematic surveys of bark beetles and ophiostomatoid fungi associated with pine have been completed in North and Central America (Zhou et al. 2004a; Kim et al. 2011; Klepzig and Hofstetter 2011; Taerum et al. 2013; Marincowitz et al. 2020), Europe (Linnakoski et al. 2012; Romón et al. 2014; Jankowiak et al. 2012, 2020), Asia (Zhou et al. 2013; Masuya et al. 2013; Kirisits et al. 2013), with a significant number of surveys conducted recently in China (Chang et al. 2017, 2019; Wang et al. 2018, 2019, 2020), South America (Zhou et al. 2004b; de Errasti et al. 2018) and New Zealand (Thwaites et al. 2005, 2013). The diversity of ophiostomatoid fungi present in exotic pine plantations in Australia, however, remains largely undetermined.

Since its first detection in the 1960s (Vaartaja 1967), *Ophiostoma ips* has been regarded as the most common fungal species associated with blue stain and pine bark beetles (specifically *Ips grandicollis*) in Australia (Stone and Simpson 1987, 1990; Hood and Ramsden 1997; Zhou et al. 2007; Carnegie et al. 2019). Additionally, surveys of the fungal associates of *Ips grandicollis* on *Pinus taeda* and *P. elliottii* in New South Wales (NSW) in the late 1980s serve as the first reports of a *Ceratocystiopsis* and *Graphilbum* species detected in Australian pine plantations (Stone and Simpson 1987, 1990), while *Grosmannia huntii* was first reported in NSW in 1998 (Jacobs et al. 1998). To date, these serve as the few detailed surveys of fungi associated with Australian pine bark beetles. Other, somewhat incidental records include the detection of *Ophiostoma floccosum, O. quercus* and an unknown species reported as a *Pesotum* aff*. fragrans*, all isolated from woodchips of *P. radiata* from the Tantanoola paper mill in South Australia (Harrington et al. 2001; Thwaites et al. 2005). Evidently, the historical record of ophiostomatoid fungi in Australian pine plantations has relied heavily on morphology, and/or the association of blue stain in the presence of the pine bark beetle, *I. grandicollis* (Carnegie and Nahrung 2019; Carnegie et al. 2019). Interestingly, the introduction of *I. grandicollis* in 1943 coincides with the introduction of two other exotic pine bark beetles, namely *Hylastes ater* and *Hylurgus ligniperda* in 1936 and 1942, respectively (Nahrung et al. 2016). Both are known to also vector ophiostomatoid fungi (Kim et al. 2011; de Errasti et al. 2018). The above-mentioned pine bark beetles, along with their associated ophiostomatoid fungi, are considered as established exotics to Australia.

Recent efforts to improve on the capacity of forest biosecurity surveillance, through programs such as the forest health surveillance program, and the more targeted high-risk site surveillance program (Carnegie et al. 2018), has led to several detections of cryptic fungal species associated with pine bark beetles and blue stain in NSW (Carnegie and Nahrung 2019). This includes the recent pest detections of *Graphilbum fragrans, O. angusticollis, O. pallidulum* and *Sporothrix cf. abietina*, illustrating the value of targeted surveillance programs for the detection of novel pests (Carnegie et al. 2019). These findings also emphasize the need for an updated record of the diversity of established ophiostomatoid fungi associated with Australian pine and pine bark beetles.

The overall aim of this study was to reconsider the diversity of ophiostomatoid fungi associated with pine and pine bark beetles in south-eastern Australia. In order to achieve this, we looked to: (1) review all available ophiostomatoid reference material previously reported from pine and lodged in Australian plant pathogen reference collections; (2) survey the ophiostomatoid fungi found in pine plantations during the 2019–2020 forest health surveillance period; and (3) use whole genome sequencing (WGS) of representative taxa to establish a curated database for improved molecular diagnostics of ophiostomatoid fungi for Australian biosecurity.

## Materials and methods

### Literature and Australian plant pathogen reference collection review

Ophiostomatoid fungi previously collected from *Pinus* spp. in Australia were included as references in this study. Living cultures were recovered from the Victorian Plant Pathology Herbarium (VPRI) and the New South Wales (NSW) Plant Pathology and Mycology Herbarium (DAR) following database searches using the currently accepted nomenclature (Seifert et al. 2013) and all putative synonyms (MycoBank Database, www.mycobank.org; Species Fungorum, www.speciesfungorum.org) of ophiostomatoid fungi that were recorded in the respective Australian collections and associated with *Pinus.* Additionally, a literature and GenBank database search (http://www.ncbi.nlm.nih.gov) was performed for Australian specimens previously reported from *Pinus* in order to identify additional specimens that had publicly available DNA sequence data.

### Sample collection during forest health surveillance

Annual forest health surveillance programs are conducted in pine plantations across Australia, including NSW (Carnegie et al. 2008), Victoria (Smith et al. 2008), Tasmania (Wotherspoon 2008), and South Australia (Phillips 2008). These surveillance programmes capture a broad overview of plantation health, achieved through aerial and ground surveys across the major growing regions for each state. Taking advantage of this routine surveillance, sampling was concentrated on pine trees showing typical symptoms of bark beetle infestation, which included any dead or dying trees, but also tree stumps in recently harvested sections. Samples were either collected and sent in by respective state agencies conducting the surveillance, or by the first author accompanying forest health surveillance. Samples collected from May 2019 to March 2020 originated from 40 locations, including collections from NSW (*n* = 34), Victoria (*n* = 2), Tasmania (*n* = 2), and South Australia (*n* = 2) (Additional file 1: Table S1). Samples of sapwood and/or pieces of bark containing beetle galleries were collected and individually placed into sampling bags to retain moisture. Where possible, pine bark beetles were collected into 50 ml collection vials on site using forceps and submitted along with their respective wood samples. Finally, all wood submissions were screened upon arrival in the laboratory for any remaining beetles that may have been concealed within the galleries. Pine bark beetles present in each sample were sorted into morphospecies, pooled and then treated as a single submission (representative specimens were morphologically identified by Crop Health Services diagnostics unit, Agriculture Victoria). All samples were stored at 4 °C until they were processed for fungal isolations.

### Fungal isolations

Fungal isolations from beetle galleries were performed by directly transferring aerial mycelia and/or spore masses found on sporing structures characteristic of ophiostomatoid fungi, such as ascomata or synnemata, onto malt extract agar (MEA; Oxoid MEA as per manufacturer instructions; Oxoid, Basingstoke, UK) amended with 0.1 g Tetracycline (Fluka Analytical, Sigma-Aldrich, MO, USA) per 1000 ml of media. When sporing structures were absent, samples were incubated in moistened plastic containers at room temperature for approximately 21 days to encourage sporulation. When blue stained sapwood was present in a sample, wood chips of approximately 5 × 5 mm were cut, surface sterilized with 1.5% sodium hypochlorite for 1 min, and plated onto MEA. Beetle isolations followed an amended protocol from Alamouti et al. (2006). Beetles from each sample were vortexed in 1 ml of 0.01% Tween80 solution (Nuplex Industries, South Australia, Australia) for 3 min. Thereafter, spore suspensions were spread onto MEA plates and incubated at 22 °C in the dark for 7 d during which all germinating single spores and hyphal tips were transferred onto individual MEA plates, producing axenic cultures which were maintained under the same growing conditions.

### Preliminary identification and ITS screening

Isolates were preliminarily grouped based on culture morphology and growth on MEA. In addition to this, a Chelex-based internal transcribed spacer (ITS) region sequencing protocol was used to confirm the putative identification of all ophiostomatoid fungi. In order to achieve this, a small amount of mycelia was scraped from each isolate using a sterile needle tip and placed into individual 200 µl reaction tubes containing 100 µl of molecular biology grade Chelex 100 resin (Bio-Rad Laboratories, Hercules, CA, USA) following a modified protocol for Chelex DNA preparation (Walsh et al. 1991). The ITS region was PCR amplified using the ITS1F and ITS4 primers (White et al. 1990; Gardes and Bruns 1993). PCR reactions included 3 µl Chelex DNA template, 15 µl of MyTaq Red mix (Bioline, London, UK), 0.4 µM of each primer (forward and reverse) and were made up to a final volume of 30 µl with nuclease free water. PCR cycle conditions followed those of Duong et al. 2012. PCR products were sent for purification and sequencing at Macrogen (Seoul, Rep. of Korea). All resulting sequences were trimmed, aligned and analysed using Geneious Prime® 2019.1.3 (www.geneious.com). Sequences were BLASTn searched against the nr/nt database of the NCBI to confirm placement within either the *Ophiostomatales* or *Microascales*. Only ophiostomatoid fungi were retained for further analysis. Finally, isolates from a given sample that shared an ITS sequence and belonged to the same morphological group were considered the same fungus, with a single axenic culture being chosen as the representative isolate in each case.

### DNA extraction, whole genome sequencing and phylogenetic analysis

Seven to 10 d old cultures were inoculated into 40 mL Potato Dextrose Broth (PDB; 9.6 g Oxoid PDB, 400 mL deionized water; Oxoid, Basingstoke, UK) and grown on a shaking incubator at 150 rpm at room temperature for approximately 72 h. Mycelia were then harvested using autoclaved Miracloth (Merck, Darmstadt, Germany) and freeze-dried before DNA extraction using the Promega Wizard Genomic DNA Purification Kit (Promega, Madison, WI, USA). The quality and quantity of extracted DNA was assessed using a Nanodrop 1000 (Thermo Fisher Scientific, MA USA) and Quantus fluorometer (Promega, Madison, WI, USA), respectively. Libraries with an average insert size of 300 bp were generated using the NextFlex Rapid XP DNA-Seq Kit (Perkin Elmer, Austin, TX, USA). Whole genome sequencing (WGS) was performed on the Novaseq 6000 system (Illumina, San Diego, CA, USA). Raw sequencing reads were quality checked and trimmed using FastP (Chen et al. 2018). Following quality trimming, initial de novo genome assemblies were produced using SPAdes v3.14.1 (Nurk et al. 2013). Assemblies were performed on error-corrected reads with a kmer range of 33, 55, 77, 97 and 111.

Assembled genomes provided a platform for sequence extraction of commonly used barcoding loci, including the ITS, the large subunit of ribosomal DNA (LSU), beta-tubulin (BT), translation elongation factor 1-α (TEF), and the calmodulin (CAL) regions. For each locus, reference sequences for type collections of ophiostomatoid fungi available in GenBank were used to create reference sets. Sequencing reads for each isolate were subsequently mapped against each reference set using BBMap (Bushnell (2014); sourceforge.net/projects/bbmap/). Locus-specific binned reads were generated for each isolate, and these reads were then mapped back to the respective de novo assembled genome in order to extract the assembled locus. This mapping step served as an additional check point to ensure cultures were axenic and only a single sequence was generated from the consensus of all mapped reads using a minimum of 10 × coverage. Extracted loci were then BLASTn searched to confirm taxonomic affinities and obtain similar sequences from GenBank to be included in phylogenetic analyses along with the sequences of type ophiostomatoid fungi.

For multi-locus phylogenetic analysis, the ITS and LSU datasets were used for initial placement of Australian isolates within well-defined species complexes of *Ophiostomatales* and *Microascales*. Subsequent phylogenetic analyses of the BT, TEF and CAL regions were performed within each species complex where loci were chosen based on availability of reference data from previous studies (e.g. BT and CAL for *Sporothrix*) which allowed for more accurate delineation of the Australian taxa. Sequence alignments were performed with MAFFT v7.388 using the E-INS-i algorithm and a gap open penalty of 1.53 (Katoh et al. 2019). The scoring matrix for alignments spanning across multiple genera was 200PAM/k = 2, while for within genus analyses the scoring matrix was set at 1PAM/k = 2 (Linnakoski et al. 2012; Katoh et al. 2019). All aligned sequence datasets were submitted to TreeBase (No. 27096). Maximum Likelihood (ML) analysis was performed with RAxML v8.2.11 (Stamatakis 2014), using the GTR model with optimization for substitution rates and the estimation of rate heterogeneity (GAMMA) specified, while the proportion of invariable sites (+ I) was selected based on results of model estimation using Smart Model Selection (SMS; Lefort et al. (2017); available at http://www.atgc-montpellier.fr/sms/). Confidence support was estimated with bootstrapping of 1000 replicates. Bayesian Inference (BI) analyses were done using MrBayes 3.2.6 (Huelsenbeck and Ronquist 2001). The substitution models and estimated rate parameters, estimated with SMS, were then included manually in MrBayes. Four Markov chain Monte Carlo (MCMC) chains were run at the same time from a random starting tree for 5 000 000 iterations. Trees were sampled every 100 generations with a burn-in length of 25%. Posterior probabilities were calculated from a majority rule consensus tree.

### Taxonomy

Morphological studies were performed on selected isolates belonging to putative novel lineages identified following phylogenetic analysis. Cultures were grown at 22 °C on 2% MEA (33 g Oxoid MEA, 10 g Oxoid agar, 1 L deionized water), as well as water agar (WA; 15 g Oxoid agar; 1 L deionized water) amended with autoclaved pine needles in order to encourage sporulation. Subsequently, reproductive structures were mounted on glass slides with 85% lactic acid and examined using Leica DM6B and M205C microscopes (Leica, Heerbrugg, Switzerland). Measurements of taxonomically characteristic structures (approximately fifty measurements for each character wherever possible) were made using a mounted Leica camera operated using the Leica application suite software v 3.06. Measurements are presented as, (minimum-) (mean-standard deviation) – (mean + standard deviation) (- maximum).

### Genomes of representative species of ophiostomatoid fungi from Australian pine plantations

Draft genomes of representative isolates for each ophiostomatoid taxon collected in this study were subjected to genome quality assessments using QUAST v5.0.2 (Mikheenko et al. 2018). In order to perform suitable comparisons, the QUAST analyses also included publicly available genomes of ophiostomatoid fungi that corresponded to the genera obtained during this study. This was done to update genome completeness assessments against the latest lineage-specific datasets available for BUSCO (Benchmarking Universal Single-Copy Orthologs tool, BUSCO; https://busco.ezlab.org/), as well as to assess gene predictions using a single prediction tool (GenMark-ES run in fungal mode). BUSCO models were predicted using the Sordariomycetes_odb10 lineage coupled with the Augustus species parameter option set as *Neurospora crassa*. Draft genome data for the representative isolates sequenced in this study has been deposited at DDBJ/EMBL/GenBank under BioProject PRJNA667796. The accession numbers for each genome are presented in Table 3.

## Results

### Sample collection and fungal isolation

A total of 135 ophiostomatoid isolates were collected during this study, 15 of which were obtained from Australian plant pathogen reference collections (Table 1). The reference isolates available from Australian collections included five *Ophiostoma ips*, five *Sporothrix* sp*.* (three of which were putatively identified as *S. cf. abietina*), two isolates residing within *Leptographium s.lat.* (one isolate, DAR 84705, identified as *Gro. huntii*), two identified as *O. angusticollis,* and a single *G. fragrans* isolate.

**Table 1:** Representative isolates of ophiostomatoid fungi associated with pine and pine bark beetles obtained during the current study

Taxon	Species	Isolate number^a,b^	Lodged as	Host^c^	Location	Collector	Year	GenBank accessions^d^				
								ITS	LSU	BT	TEF	CAL
*Ophiostomatales*												
1	*Ceratocystiopsis* sp.	VPRI43766		*Pr*	Moss Vale, NSW	Carnegie, A. J	2019	MW046061	MW046107	MW066349	MW066395	MW075110
		VPRI43834		*Pp*	Whiporie, NSW	Carnegie, A. J., Trollip, C	2019	MW046062	MW046108	MW066350	MW066396	MW075111
		VPRI43835		*Pcxe*	Bonalbo, NSW	Carnegie, A. J., Trollip, C	2019	MW046063	MW046109	MW066351	MW066397	MW075112
		VPRI43836		*Pt*	Urbenville, NSW	Carnegie, A. J., Trollip, C	2019	MW046064	MW046110	MW066352	MW066398	MW075113
2	*Graphilbum fragrans*	DAR84707^H^ (VPRI43528)	*G. fragrans*	*Pr*	Vittoria, NSW	Carnegie, A. J	2018	MW046065	MW046111	MW066353	MW066399	MW075114
		VPRI43756		*Pr*	Vittoria, NSW	Sargeant, D	2019	MW046066	MW046112	MW066354	MW066400	MW075115
		VPRI43758		*Pr*	Rockley, NSW	Sargeant, D	2019	MW046067	MW046113	MW066355	MW066401	MW075116
3	***G. ipis-grandicollis***	VPRI43759		*Pr*	Inverell, NSW	Carnegie, A. J	2019	MW046068	MW046114	MW066356	MW066402	MW075117
		VPRI43760		*Pr*	Tumut, NSW	Sargeant, D	2019	MW046069	MW046115	MW066357	MW066403	MW075118
		VPRI43761^M^		*Pr*	Moss Vale, NSW	Carnegie, A. J	2019	MW046070	MW046116	MW066358	MW066404	MW075119
		VPRI43762™		*Pr*	Moss Vale, NSW	Carnegie, A. J	2019	MW046071	MW046117	MW066359	MW066405	MW075120
4	*G. cf. rectangulosporium*	VPRI43763		*Pr*	Rosewood, NSW	Carnegie, A. J	2019	MW046072	MW046118	MW066360	MW066406	MW075121
		VPRI43843		*Pt*	Urbenville, NSW	Carnegie, A. J., Trollip, C	2019	MW046073	MW046119	MW066361	MW066407	MW075122
5	*Grosmannia huntii*	DAR84705^H^ (VPRI43530)	*G. huntii*	*Pr*	Bombala, NSW	Carnegie, A. J	2018	MW046074	MW046120	MW066362	MW066408	MW075123
		VPRI22395^H^	*Leptographium* sp.	*Pr*	South Yarra, VIC	Smith, I	2000	MW046075	MW046121	MW066363	MW066409	MW075124
		VPRI43837		*Pr*	Lower Beulah, TAS	Wotherspoon, K., Ramsden, N	2020	MW046076	MW046122	MW066364	MW066410	MW075125
6	*Gro. radiaticola*	VPRI43523		*Pr*	Nangwarry, SA	Smith, D	2019	MW046077	MW046123	MW066365	MW066411	MW075126
		VPRI43838		*Pr*	Branxholm, TAS	Wotherspoon, K., Ramsden, N	2020	MW046078	MW046124	MW066366	MW066412	MW075127
		VPRI43839		*Pr*	Lower Beulah, TAS	Wotherspoon, K., Ramsden, N	2020	MW046079	MW046125	MW066367	MW066413	MW075128
7	*Ophiostoma angusticollis*	VPRI43764^H^	*O. angusticollis*	*Pr*	Rosewood, NSW	Sargeant, D	2019	MW046080	MW046126	MW066368	MW066414	MW075129
		VPRI43765^H^	*O. angusticollis*	*Pr*	Vittoria, NSW	Sargeant, D	2019	MW046081	MW046127	MW066369	MW066415	MW075130
8	*O. fasciatum*	VPRI43845		*Pcxe*	Whiporie, NSW	Carnegie, A. J	2019	MW046082	MW046128	MW066370	MW066416	MW075131
9	*O. ips*	DAR84692^H^ (VPRI43529)	*O. ips*	*Pr*	Neville, NSW	Carnegie, A. J	2017	MW046083	MW046129	MW066371	MW066417	MW075132
		DAR84817^H^ (VPRI43861)	*O. ips*	*Pr*	Tumbarumba, NSW	Carnegie, A. J	2018	MW046084	MW046130	MW066372	MW066418	MW075133
		VPRI43316^H^	*Ophiostoma* sp.	*Pr*	Chiltern, VIC	Smith, D	2018	MW046085	MW046131	MW066373	MW066419	MW075134
		VPRI42284^H^	*Ophiostoma* sp.	*Pr*	Shelley, VIC	Smith, D	2013	MW046086	MW046132	MW066374	MW066420	MW075135
		VPRI42255^H^	*O. ips*	*Pr*	Shelley, VIC	Smith, D	2013	MW046087	MW046133	MW066375	MW066421	MW075136
		VPRI43731		*Pr*	Moss Vale, NSW	Carnegie, A. J	2019	MW046088	MW046134	MW066376	MW066422	MW075137
		VPRI43734		*Pcxe*	Urbenville, NSW	Sargeant, D	2019	MW046089	MW046135	MW066377	MW066423	MW075138
		VPRI43738		*Pr*	Tumut, NSW	Sargeant, D	2019	MW046090	MW046136	MW066378	MW066424	MW075139
		VPRI43743		*Pr*	Batlow, NSW	Sargeant, D	2019	MW046091	MW046137	MW066379	MW066425	MW075140
		VPRI43851		*Pr*	Urbenville, NSW	Carnegie, A. J., Trollip, C	2019	MW046092	MW046138	MW066380	MW066426	MW075141
10	*O. pallidulum*	VPRI43846		*Pr*	Lower Beulah, TAS	Wotherspoon, K., Ramsden, N	2020	MW046093	MW046139	MW066381	MW066427	MW075142
11	*Raffaelea deltoideospora*	VPRI43720		*Pcxe*	Urbenville, NSW	Sargeant, D	2019	MW046094	MW046140	MW066382	MW066428	MW075143
12	*Sporothrix euskadiensis*	VPRI43754		*Pr*	Batlow, NSW	Sargeant, D	2019	MW046095	MW046141	MW066383	MW066429	MW075144
13	*S. cf. nigrograna*	VPRI43755		*Pr*	Bathurst, NSW	Sargeant, D	2019	MW046096	MW046142	MW066384	MW066430	MW075145
14	*S. pseudoabietina*	DAR84706^H^ (VPRI43531)	*S. cf. abietina*	*Pr*	Batlow, NSW	Carnegie, A. J	2019	MW046097	MW046143	MW066385	MW066431	MW075146
		DAR84897^H^ (VPRI43867)	*Sporothrix* sp*.*	*Pcxe*	Whiporie, NSW	Carnegie, A. J	2019	MW046098	MW046144	MW066386	MW066432	MW075147
		DAR84898^H^ (VPRI43868)	*O. nigrocarpum*	*Pcxe*	Whiporie, NSW	Carnegie, A. J	2019	MW046099	MW046145	MW066387	MW066433	MW075148
		DAR84899^H^ (VPRI43869)	*O. nigrocarpum*	*Pcxe*	Whiporie, NSW	Carnegie, A. J	2019	MW046100	MW046146	MW066388	MW066434	MW075149
		DAR84900^H^ (VPRI43870)	*Sporothrix* sp*.*	*Pcxe*	Whiporie, NSW	Carnegie, A. J	2019	MW046101	MW046147	MW066389	MW066435	MW075150
		VPRI43721		*Pr*	Benalla, VIC	Smith, D., Trollip, C	2019	MW046102	MW046148	MW066390	MW066436	MW075151
		VPRI43749		*Pcxe*	Urbenville, NSW	Sargeant, D	2019	MW046103	MW046149	MW066391	MW066437	MW075152
		VPRI43751		*Pcxe*	Urbenville, NSW	Sargeant, D	2019	MW046104	MW046150	MW066392	MW066438	MW075153
		VPRI43752		*Pcxe*	Whiporie, NSW	Sargeant, D	2019	MW046105	MW046151	MW066393	MW066439	MW075154
*Microascales*												
15	*Graphium *sp.	VPRI43844		*Pe*	Whiporie, NSW	Carnegie, A. J., Trollip, C	2019	MW046106	MW046152	MW066394	MW066440	MW075155

The species name printed in bold type represent novel taxa

^a^The Victorian Plant Pathology Herbarium (VPRI); The NSW Plant Pathology and Mycology Herbarium (DAR)

^b^H = Isolate obtained from plant pathogen reference collection; T = ex-holotype isolate; M = isolates used for morphological study

^c^Host: *Pr, Pinus radiata*; *Pp, P. ponderosa; Pcxe, P. caribaea* x *elliottii; Pt, Pinus taeda; Pe, P. elliottii*

^d^ITS, The internal transcribed spacer; LSU, The large ribosomal subunit (28S); BT, β-tubulin; TEF, Translation elongation factor 1-α; CAL, Calmodulin

The remaining 120 isolates were obtained from samples received during the 2019–20 forest health surveillance period, which included isolations from beetles, beetle galleries and blue-stained wood chips (Additional file 1: Table S1). Samples were largely collected from *Pinus radiata* (62.5%), the most common *Pinus* species grown across temperate regions of south-eastern Australia, and *P. caribaea* x *elliottii* hybrids (22.5%), the most commonly planted species in the subtropical parts of northern NSW. The remainder were collected from *P. taeda* (7.5%), *P. elliottii* (5%), and included a single sample from an amenity planting of *P. ponderosa*. Three species of pine bark beetles, namely *Ips grandicollis, Hylastes ater* and *Hylurgus ligniperda,* and the ambrosia beetle *Xyleborus* nr. *ferrugineus.,* were recovered from 22 of the samples collected (Additional file 1: Table S1). *Ips grandicollis* was the most abundant beetle species sampled during this study, comprising approximately 97% of the beetles included in our dataset. Samples containing *H. ater* and *Hy. ligniperda* came only from sites in South Australia and Tasmania respectively, while a single sample from northern NSW included the *Xyleborus* species.

Preliminary identification and ITS screening characterised the 120 ophiostomatoid isolates into 15 taxonomic groups, 14 of which resided in , and a single taxon belonged to *Microascales* (Table 1). Ophiostomatoid isolates were recovered evenly from the sampled pine tissue (56%) and bark beetles (44%), with about two thirds of all isolations associated with a *P. radiata* host (Additional file 2: Table S2). *Ophiostoma ips* (Taxon 9) and *Sporothrix pseudoabietina* (Taxon 14) were isolated most frequently, making up approximately 53% and 19% of the dataset, respectively (Additional file 2: Table S2). This trend was consistent for the abundantly sampled bark beetle vector, *Ips grandicollis,* where five additional taxa (taxa 1, 3, 4, 8 and 15) were represented by the 44 fungal isolates collected from this source. The remaining taxa were only recovered occasionally, with the host association and isolation frequencies recorded in Additional file 2: Table S2. Finally, 46 isolates representing all major taxonomic groups were selected for further phylogenetic analysis and taxonomic placement (Table 1).

### Phylogenetic analysis

**Fig. 1:**
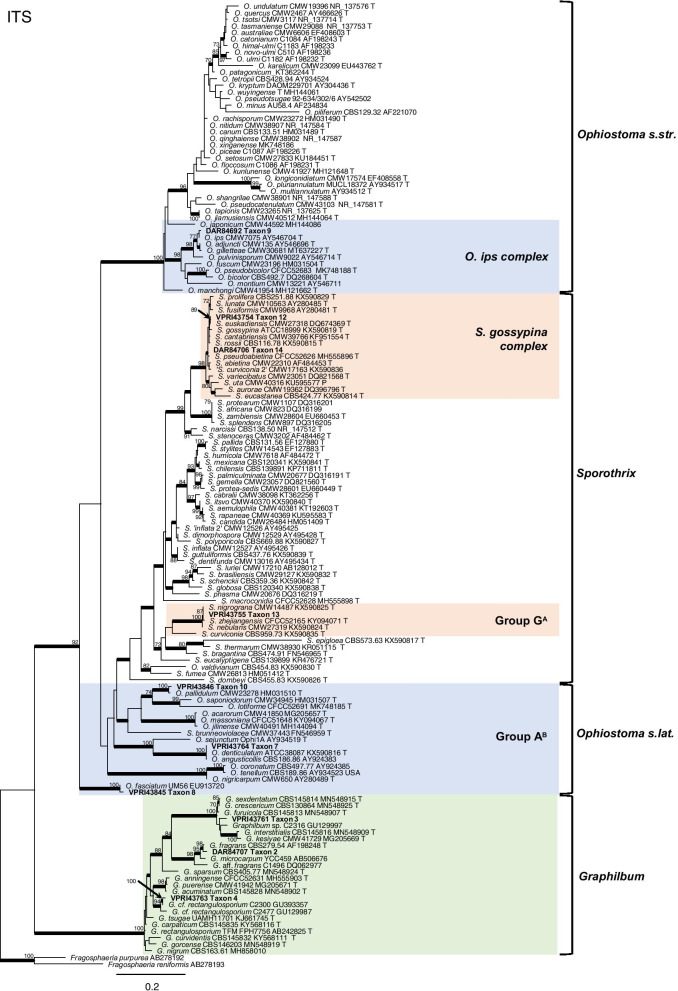
ML phylogeny of the ITS region for isolates residing in *Ophiostoma*, *Sporothrix*. and *Graphilbum*. Sequences generated in this study are printed in bold. Bold branches indicate posterior probability values ≥ 0.9, while ML bootstrap values of ≥ 70% are recorded at nodes. T = ex-type cultures. A Group name as described by de Beer et al. (2016). B Group name as described by Chang et al. (2017)

**Fig. 2:**
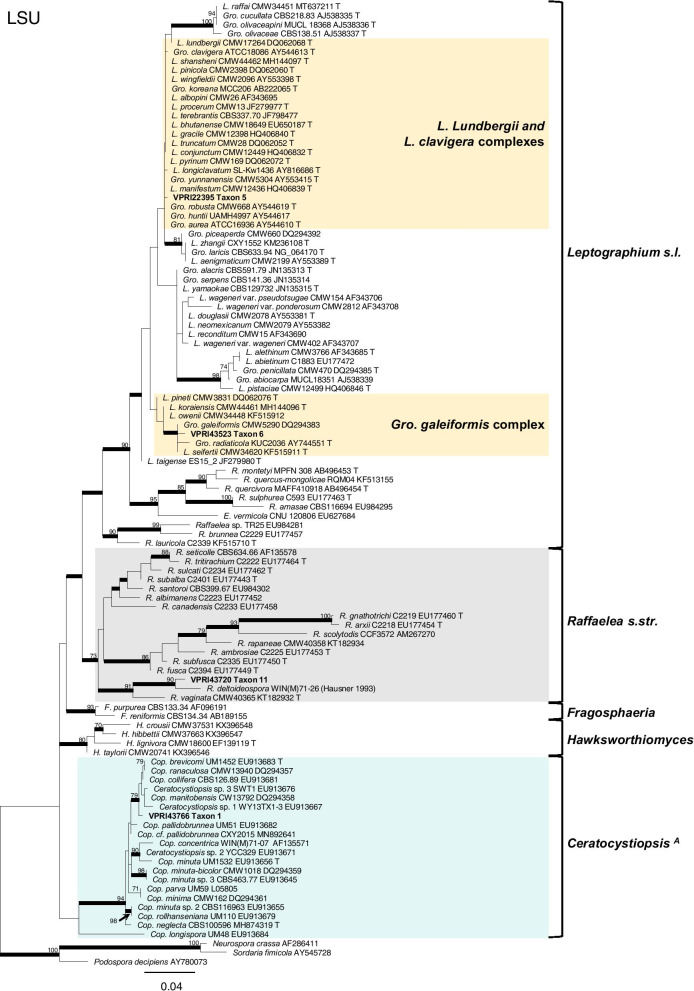
ML phylogeny of LSU region for isolates residing in *Ceratocystiopsis*, *Leptographium s. lat*. and *Raffaelea*. Sequences generated in this study are printed in bold. Bold branches indicate posterior probability values ≥ 0.9, while ML bootstrap values of ≥ 70% are recorded at nodes. T = ex-type cultures. A Taxon names as described by de Beer and Wingfield (2013)

**Fig. 3:**
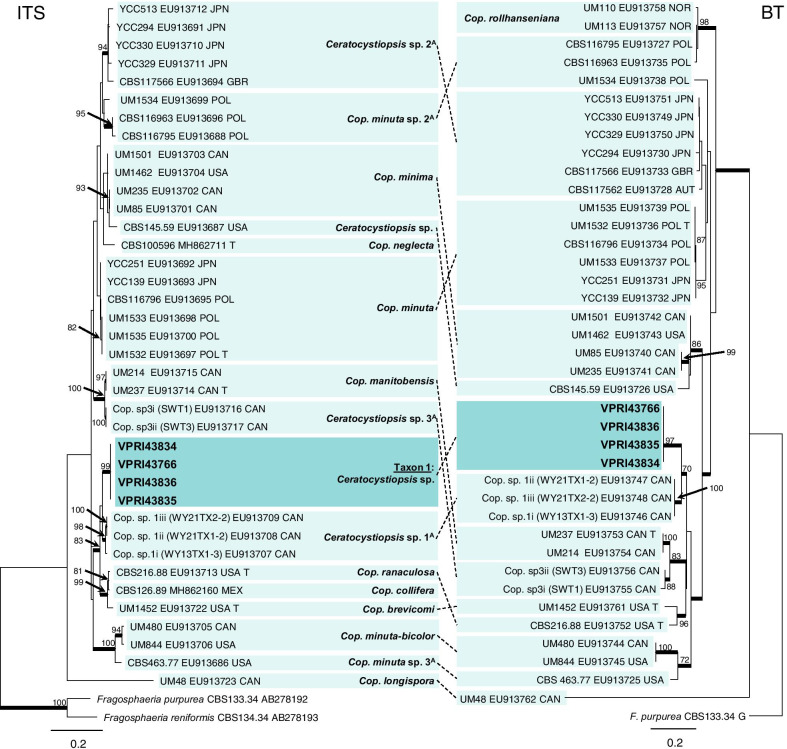
Phylogenetic analysis of isolates residing in *Ceratocystiopsis.* Sequences generated in this study are printed in bold. Bold branches indicate posterior probability values ≥ 0.9, while ML bootstrap values of ≥ 70% are recorded at nodes. T = ex-type isolates, G = sequence retrieved from genome. ^A^Current taxon name as described by De Beer and Wingfield (2013)

**Fig. 4:**
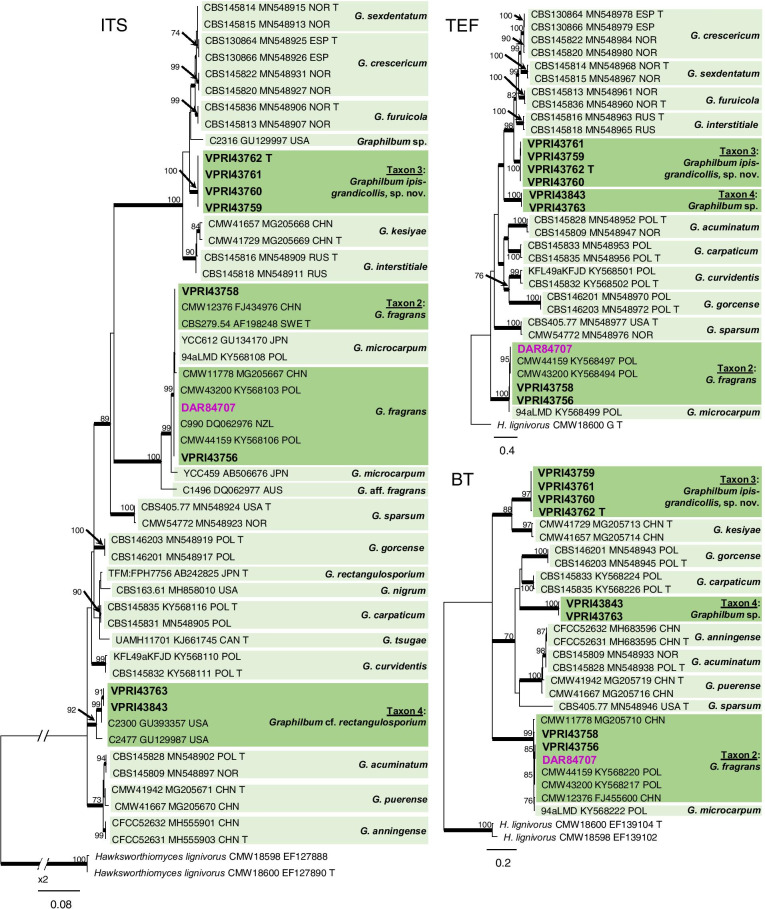
Phylogenetic analysis of isolates residing in *Graphilbum.* Sequences generated in this study are printed in bold with reference collection isolates coloured purple. Bold branches indicate posterior probability values ≥ 0.9, while ML bootstrap values of ≥ 70% are recorded at nodes. T = ex-type cultures, G = sequence retrieved from genome

**Fig. 5:**
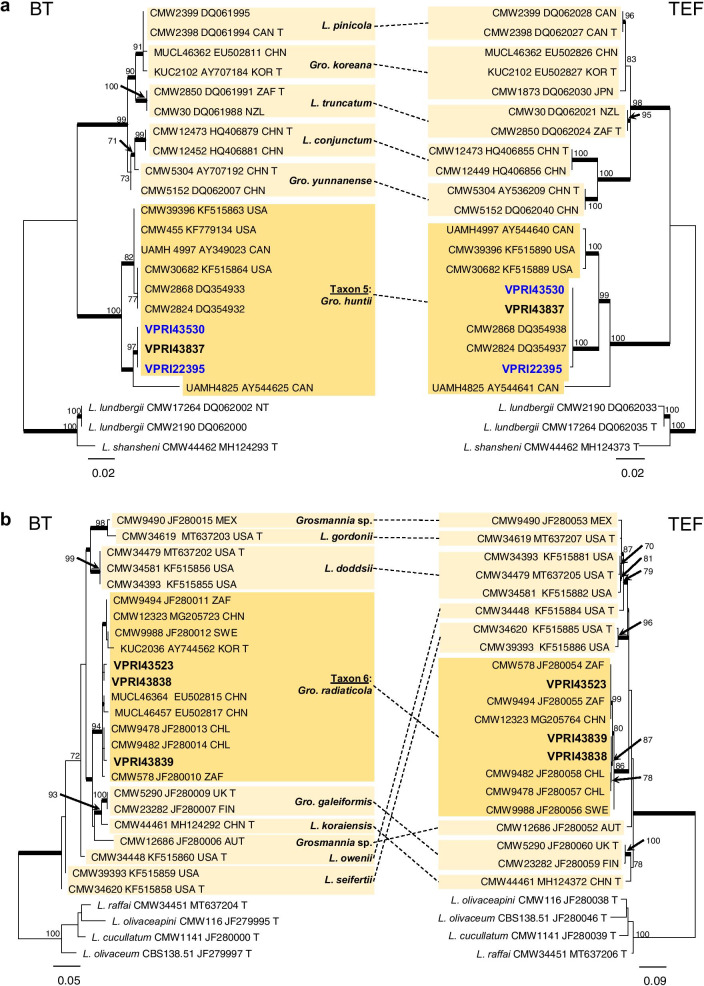
Phylogenetic analysis of BT and TEF for *Leptographium s.lat.* **a.** Isolates residing in the *L. lundbergii* and *Gro. huntii* species complexes. **b.** Isolates residing in the *Gro. galeiformis* species complex. Sequences generated in this study are printed in bold, with reference collection isolates coloured blue. Bold branches indicate posterior probability values ≥ 0.9, while ML bootstrap values of ≥ 70% are recorded at nodes. T = ex-type cultures

**Fig. 6:**
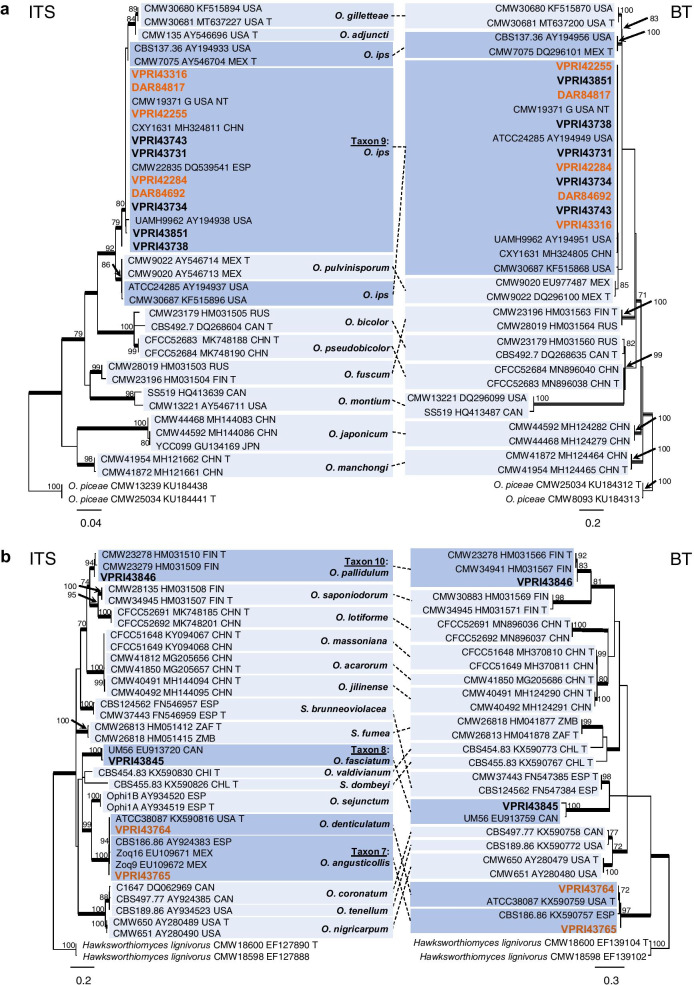
Phylogenetic analysis of ITS and BT for isolates residing in *Ophiostoma.* **a.** Analysis of isolates belonging in the *O. ips* complex. **b.** Analysis of isolates belonging in ‘Group A’*.* Sequences generated in this study are printed in bold, with reference collection isolates coloured orange. Bold branches indicate posterior probability values ≥ 0.9, while ML bootstrap values of ≥ 70% are recorded at nodes. T = ex-type cultures

**Fig. 7:**
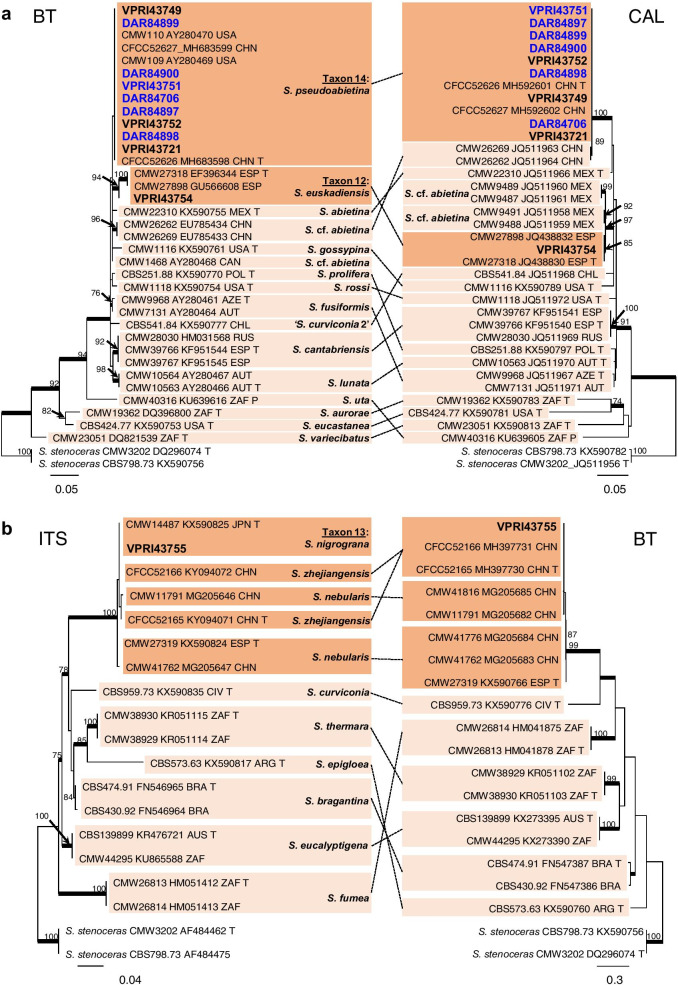
Phylogenetic analysis of isolates residing in *Sporothrix*. **a.** Analysis of the BT and CAL regions for the *S. gossypina* complex. **b.** Analysis of the ITS and BT regions for *Sporothrix ‘Group G’.* Sequences generated in this study are printed in bold, with reference collection isolates coloured blue. Bold branches indicate posterior probability values ≥ 0.9. ML bootstrap values of ≥ 70% are recorded at nodes. T = ex-type cultures

**Fig. 8:**
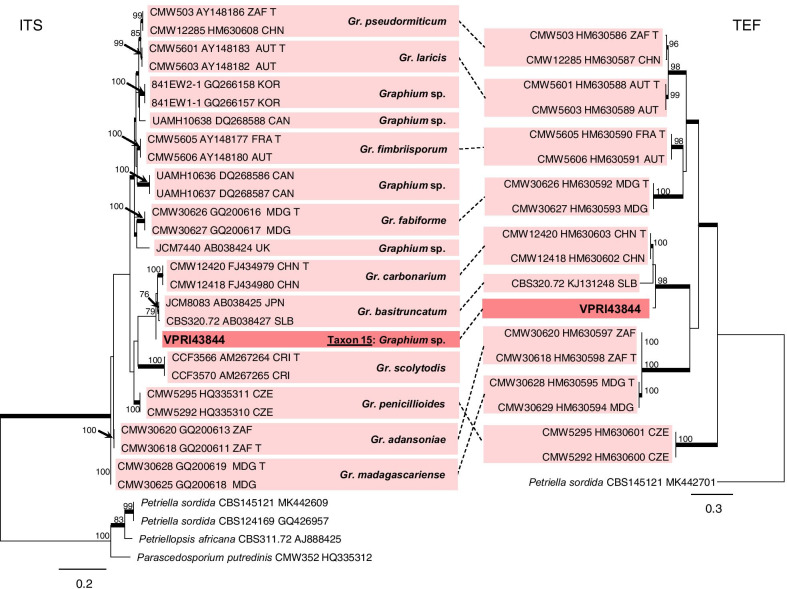
ML phylogenies of *Graphium* isolates generated from ITS and TEF sequence data. Sequences generated in this study are printed in bold type. Bold branches indicate posterior probability values ≥ 0.9. ML bootstrap values of ≥ 70% are recorded at nodes. T = ex-type cultures

Phylogenetic analysis of the ITS (Fig. 1) and LSU (Fig. 2) regions allowed for taxa to be sorted into their respective species complexes, while the additional gene regions of BT, TEF and CAL (Figs. 3, 4, 5, 6, 7, 8, Additional files 4 and 5: Fig. S1, S2) enabled species level resolution and more accurate delineation. In *Ophiostomatales*, the 14 taxonomic groups were found to encompass six genera: *Ceratocystiopsis* (Taxon 1)*, Graphilbum* (Taxa 2–4)*, Leptographium s. lat.* (Taxa 5–6)*, Ophiostoma s. lat.* (Taxa 7–10), *Raffaelea* (Taxon 11), and *Sporothrix* (Taxa 12–14). The single taxon residing in *Microascales* was identified as belonging to *Graphium* (Taxon 15).

#### Ophiostomatales

Taxon 1 comprised of four representative isolates grouping as a well-supported clade within *Ceratocystiopsis* (Figs. 2, 3). Phylogenetic analysis of the LSU dataset revealed Taxon 1 grouped as an independent lineage, close to *Ceratocystiopsis* (*Cop.*) *ranaculosa* and *Cop. brevicomis* (Fig. 2). Analysis of ITS and BT regions (Fig. 3) supports this placement and illustrates that the Australian isolates are most closely related to a previously undescribed taxon reported as *Ceratocystiopsis* species 1 (*Cop*. *minuta*-like) from Canada (Plattner et al. 2009)*.* Multi-locus analysis suggests the isolates of *Ceratocystiopsis* sp. (Taxon 1) represent a novel lineage*.*

Three taxa (Taxa 2, 3 and 4) residing within *Graphilbum* were collected during this study (Fig. 1). Reference collection isolate DAR84707 and two representative isolates collected during this survey forming Taxon 2 (Table 1) were confirmed as *Graphilbum fragrans* (Figs. 1, 4). Taxon 3 and 4 (which comprised of four and two isolates, respectively; Table 1) were both preliminarily identified as *G. cf. rectangulosporium* isolates, with BLASTn searches suggesting an affiliation to previously reported isolates from the USA, China, and Europe. Further analysis of the BT, TEF and CAL regions (Figs. 1, 4, Additional file 4: Fig. S1) revealed that Taxon 3 represented a phylogenetically distinct lineage, forming part of a species complex including *G. crescericum, G. furuicola*, *G. interstitiale*, *G. kesiyae*, and *G. sexdentatum*. This new species is described below. Taxon 4 formed a well-supported clade with two previously undescribed *Graphilbum* isolates reported from the USA (Fig. 4).

Within *Leptographium s.l.,* two taxa (Taxa 5 and 6) were collected (Table 1; Fig. 2). Taxon 5 comprised of two reference collection isolates, as well as a single isolate collected during this survey. Phylogenetic analysis confirmed Taxon 5 as *Grosmannia huntii* (Figs. 2, 5a). Taxon 6 comprised of three representative isolates which fell into a clade within the *Gro. galeiformis* species complex (Fig. 2). Analysis of BT and TEF confirmed the delineation of Taxon 6 as the species *Gro. radiaticola* (Fig. 5b).

Taxa 7 to 10 resided within *Ophiostoma s. lat.* with Taxon 9 the only one belonging to a well-recognised species complex (Fig. 1). Taxon 9 included the reference collection isolates DAR84692, DAR84817, VPRI42284, VPRI42255 and VPRI43316, along with five additional isolates collected during this study (Table 1). Analysis of the ITS region identified Taxon 9 isolates as *Ophiostoma ips* (Fig. 1). Despite incongruence with regards to the delimitation of *O. ips* using ITS alone, BT analysis confirmed little variation between the Australian isolates, and established a clear grouping with several isolates recently confirmed as *O. ips* (Fig. 6a). Taxa 7, 8 and 10 all grouped peripherally to *Ophiostoma s. str*. and are regularly referred to as Group A/Lineage G (Chang et al. 2017; Wang et al. 2020). Taxon 7 included two reference collection isolates forming a lineage along with two species, namely *O. angusticollis* and *O. denticulatum* (Fig. 6b). The currently available molecular data for reference specimens within this lineage is lacking for appropriate taxonomic comparison, and thus clear differentiation between these species is limited. For now, Taxon 7 is referred to as *O. angusticollis*. Taxon 8 included a single isolate collected during this study (Table 1), with ITS and BT analyses identifying this taxon as *O. fasciatum* (Figs. 1, 6b). Taxon 10 included a single strain preliminarily identified as *O. pallidulum* (Fig. 1). BT analysis further confirmed this identification (Fig. 6b).

The single isolate of Taxon 11 grouped within *Raffaelea s. str.* (Fig. 2). Analysis of the LSU sequence showed that the Australian isolate forms a well-supported lineage with *Raffaelea deltoideospora* (Fig. 2)*.* Analysis of the ITS region further validated Taxon 11’s placement within *Raffaelea s. str*. and the delimitation of this species as *R. deltoideospora* (Additional file 5: Fig. S2).

Three *Sporothrix* taxa were obtained during this study (Taxa 12, 13, and 14; Table 1). Taxon 12 and Taxon 14 both grouped within the *S. gossypina* complex (Fig. 1). While analysis of the ITS region gave limited resolution within the *S. gossypina* complex (Fig. 1), analysis of BT and CAL was able to distinguish between the closely related species (Fig. 7a). The single isolate of Taxon 12 was identified as *S. euskadiensis* (Romón et al. 2014). Taxon 14 included all reference collection *Sporothrix* isolates as well as 5 additional representative isolates collected during this study (Table 1). ML analysis placed isolates of Taxon 14 in a well-supported clade alongside the type strain of *S. pseudoabietina* (Fig. 7a). Taxon 13 comprised of a single isolate collected in this study, with the ITS region placing the taxon among species belonging to a group within *Sporothrix* recently referred to as “Group G” (De Beer et al. 2016) (Fig. 1). Taxon 13 shared an almost identical ITS sequence with the type sequences for *S. nigrograna* and *S. zhejiangensis* (Fig. 7b). While analysis of the BT region was unable to clearly distinguish between *S. nebularis* and *S. zhejiangensis* (Fig. 7b), analysis of CAL did show good support for the distinction of Taxon 13 from *S. nebularis* (Additional file 6: Fig. S3)*.* A lack of available molecular data for these species limited further phylogenetic comparisons and thus, the placement of the Australian taxon.

#### Microascales

The single isolate (Taxon 15) residing in *Microascales* was identified as a *Graphium* species (Fig. 8). Analysis of ITS and TEF regions revealed that this isolate resides closely to the species of *Gra*. *basitruncatum* and *Gra. carbonarium* (Fig. 8). While this taxon may represent a novel lineage, we have chosen not to formally describe it until additional specimens and/or reference material can be examined. Taxon 15 is thus referred to as a *Graphium* species.

#### Species of Ophiostomatales and Microascales associated with Australian Pinus , verified by DNA sequence data

Revision of the literature, as well as the database search, allowed for the comparison of our results to the historical records of ophiostomatoid fungi associated with *Pinus* in Australia (Table 2). While several taxa identified in the current study represent first records for Australia, molecular sequence data has verified the previous morphological records of a *Ceratocystiopsis* sp. (Stone and Simpson 1987) and a *Graphium* sp. (Vaartaja 1967). Notably however, five previous records still require molecular confirmation and their current status should be treated with care due to the numerous taxonomic re-evaluations that have taken place since their initial identification (Table 2; footnotes).

**Table 2:** Current status list of Australian ophiostomatoid fungi associated with *Pinus*

Genus	Species/taxon recorded	State/s	Verified	GenBank accession	References
*Ophiostomatales*					
*Ceratocystiopsis*	***Ceratocystiopsis*** sp.^a^	NSW	+	Table 1	Stone and Simpson (1987, 1990), Current study
	*Ceratocystiopsis minuta*	NSW	−	NA	Stone and Simpson (1990)
*Graphilbum*^b^	*Pesotum* aff. *fragrans*	SA	+	DQ062977	Harrington et al. (2001); Thwaites et al. (2005)
	***Graphilbum fragrans***	NSW	+	Table 1	Carnegie et al. (2019), Current study
	***Graphilbum ipis-grandicollis, ***sp. nov.	NSW	+	Table 1	Current study
	***Graphilbum*cf.*rectangulosporium***	NSW	+	Table 1	Current study
*Ophiostoma*	*Ophiostoma pilifera*	VIC	−		Eckersley (1934), Rawlings (1960)
	***Ophiostoma ips***	NSW, QLD, SA, VIC	+	Table 1	Vaartaja 1967, Zhou et al. 2007, Carnegie et al. 2019, Current study
	*Ophiostoma floccosum*	SA	−	NA	Harrington et al. (2001)
	*Ophiostoma quercus*	SA	−	NA	Harrington et al. (2001)
	***Ophiostoma angusticollis***	NSW	+	Table 1	Carnegie et al. (2019), Current study
	***Ophiostoma pallidulum***	NSW, TAS	+	Table 1	Carnegie et al. (2019) Current study
	***Ophiostoma fasciatum***	NSW	+	Table 1	Current study
*Leptographium s.l*	***Grosmannia huntii***	NSW, VIC, TAS	+	Table 1	Jacobs et al. (1998), Carnegie et al. (2019), Current study
	*Leptographium* sp.^c^	TAS	−	NA	Griggs, J.A. thesis (1998)
	***Grosmannia radiaticola***	SA, TAS	+	Table 1	Current study
*Raffaelea*	***Raffaelea deltoideospora***	NSW	+	Table 1	Current study
*Sporothrix*	***Sporothrix pseudoabietina***	NSW, VIC	+	Table 1	Carnegie et al. (2019), Current study
	***Sporothrix euskadiensis***	NSW	+	Table 1	Current study
	***Sporothrix*cf.*nigrograna***	NSW	+	Table 1	Current study
*Microascales*					
*Graphium*^d^	***Graphium*sp.**	NSW	+	Table 1	Vaartaja (1967)

Species identified in the current study are presented in bold, and their accession details can be found in Table 1

^a^We speculate that the taxon reported as *Ceratocystiopsis* sp. by Stone and Simpson (1987, 1990) is likely the same taxon recorded in the current study. Refer to discussion for more information

^b^Stone and Simpson (1990) reported a *Graphilbum* sp. associated with *Ips grandicollis* in NSW. This taxon could refer to any of the four taxa currently confirmed using molecular data

^c^Griggs, J.A. recorded *Leptographium lundbergii* in association with *Hylastes ater* infesting *P*. *radiata* in TAS. This ID has not been verified molecularly and should be treated with caution considering the taxonomic re-evaluation of *L. lundbergii* by Jacobs et al. (1998)

^d^Vaartaja (1967) identified several species of *Graphium*. This descriptor is somewhat ambiguous and could refer to species in both the *Ophiostomatales* and *Microascales*

### Draft genomes of representative isolates of ophiostomatoid fungi from Australian pine plantations

Genome summary statistics of the representative draft genomes produced in this study are summarised in Table 3 (see Additional file 3: Table S3 for extended comparison). Genomes were assembled to an average size of 28 Mb and were represented by a mean scaffold number of 148. The N50 ranged from 208,570 to 1,285,428 bp, with the longest contig of 3,412,636 bp generated for the *S. pseudoabietina* strain, VPRI34531. The GC content had a mean of 57%, with a standard deviation of 3% from this mean. Gene predictions resulted in an average estimate of 7800 Open Reading Frames (ORFs), with a gene density ranging from 240 to 341 ORFs/Mb. All draft genomes had a high BUSCO completeness assessment score ranging between 93.48 and 98.24%. All representative draft genomes were made publicly available on GenBank with Accession details summarised in Table 3.

**Table 3:** Genome summary statistics of representative ophiostomatoid isolates sequenced in this study

Species	*Ceratocystiopsis*	*Graphilbum*			*Leptographium s. lat.*	
	*Ceratocystiopsis* sp.	*G. fragrans*	*G. ipis-grandicollis* sp. nov	*G. cf. rectangulosporium*	*Gro. huntii*	*Gro. radiaticola*
Taxon	Taxon 1	Taxon 2	Taxon 3	Taxon 4	Taxon 5	Taxon 6
Sequenced strain	VPRI43766	VPRI43528	VPRI43762	VPRI43763	VPRI43530	VPRI43523
GenBank Accession	JADHKF010000000	JADHKG010000000	JADHKH010000000	JADHKI010000000	JADHKJ010000000	JADHKK010000000
Total reads after QC	27,063,242	50,135,289	49,429,518	46,778,484	94,294,776	114,599,394
Number of scaffolds	79	237	178	117	254	85
Longest contig (bp)	1,540,000	999,956	1,555,217	1,343,814	1,099,032	2,583,828
Est. genome size (Mb)	20.45	34.04	24.02	23.61	28.05	27.56
N50 (bp)	471,680	323,198	601,306	298,270	343,842	883,760
L50	12	32	13	22	25	11
# N's per 100 kbp	10	7	14	6	8	5
GC (%)	61.48	55.75	55.53	60.91	54.63	57.09
Avg. coverage depth	197	218	305	296	493	619
No. of predicted genes	6967	9034	7221	7270	7836	7867
Est. gene density	341	265	301	308	279	286
Complete BUSCO (%)	95.20	97.10	95.60	95.70	97.40	96.40
Complete BUSCO (n)	3636	3706	3647	3651	3716	3680
Complete—single	3631	3699	3644	3648	3710	3673
Complete—duplicated	5	7	3	3	6	7
Fragmented	17	23	29	18	17	21
Missing	164	88	141	148	84	116

Species	*Ophiostoma s. lat.*			*Raffaelea*	*Sporothrix*			*Graphium*
	*O. fasciatum*	*O. ips*	*O. pallidulum*	*R. deltoideospora*	*S. euskadiensis*	*S. cf. nigrograna*	*S. pseudoabietina*	*Graphium *sp.
Taxon	Taxon 8	Taxon 9	Taxon 10	Taxon 11	Taxon 12	Taxon 13	Taxon 14	Taxon 15
Sequenced strain	VPRI43845	VPRI43529	VPRI43846	VPRI43720	VPRI43754	VPRI43755	VPRI43531	VPRI43844
GenBank Accession	JADHKM010000000	JADHKN010000000	JADHKO010000000	JADHKP010000000	JADHKQ010000000	JADHKR010000000	JADHKS010000000	JADHKT010000000
Total reads after QC	51,287,508	37,261,588	36,666,452	35,927,882	29,627,312	37,781,794	36,719,768	28,160,580
Number of scaffolds	36	208	473	120	92	125	51	490
Longest contig (Mb)	2,360,849	1,117,630	543,815	1,965,135	1,881,930	1,307,617	3,412,636	1,008,604
Est. genome size (Mb)	22.54	26.01	32.66	30.95	36.13	27.34	35.20	31.65
N50 (bp)	966,108	283,044	126,120	416,364	790,753	398,989	1,285,428	190,968
L50	9	29	84	25	16	23	9	49
# N's per 100 kbp	4	8	12	18	11	12	8	12
GC (%)	65.26	56.88	57.93	54.55	52.70	59.54	53.27	50.12
Avg. coverage depth	341	209	167	173	122	205	154	132
No. of predicted genes	7498	7470	8757	7428	9348	7908	9190	9482
Est. gene density	333	287	268	240	259	289	261	300
Complete BUSCO (%)	96.60	96.90	97.20	94.90	98.10	97.20	97.80	96.60
Complete BUSCO (n)	3688	3696	3710	3621	3743	3710	3734	3686
Complete—single	3685	3690	3705	3613	3739	3703	3729	3679
Complete—duplicated	3	6	5	8	4	7	5	7
Fragmented	16	16	28	30	10	23	12	40
Missing	113	105	79	166	64	84	71	91

## Taxonomy

***Graphilbum ipis-grandicollis*** C. Trollip, Q. Dinh, & Jacqueline Edwards, **sp. nov.**

MycoBank: MB840696.

(Fig. 9)

**Fig. 9:**
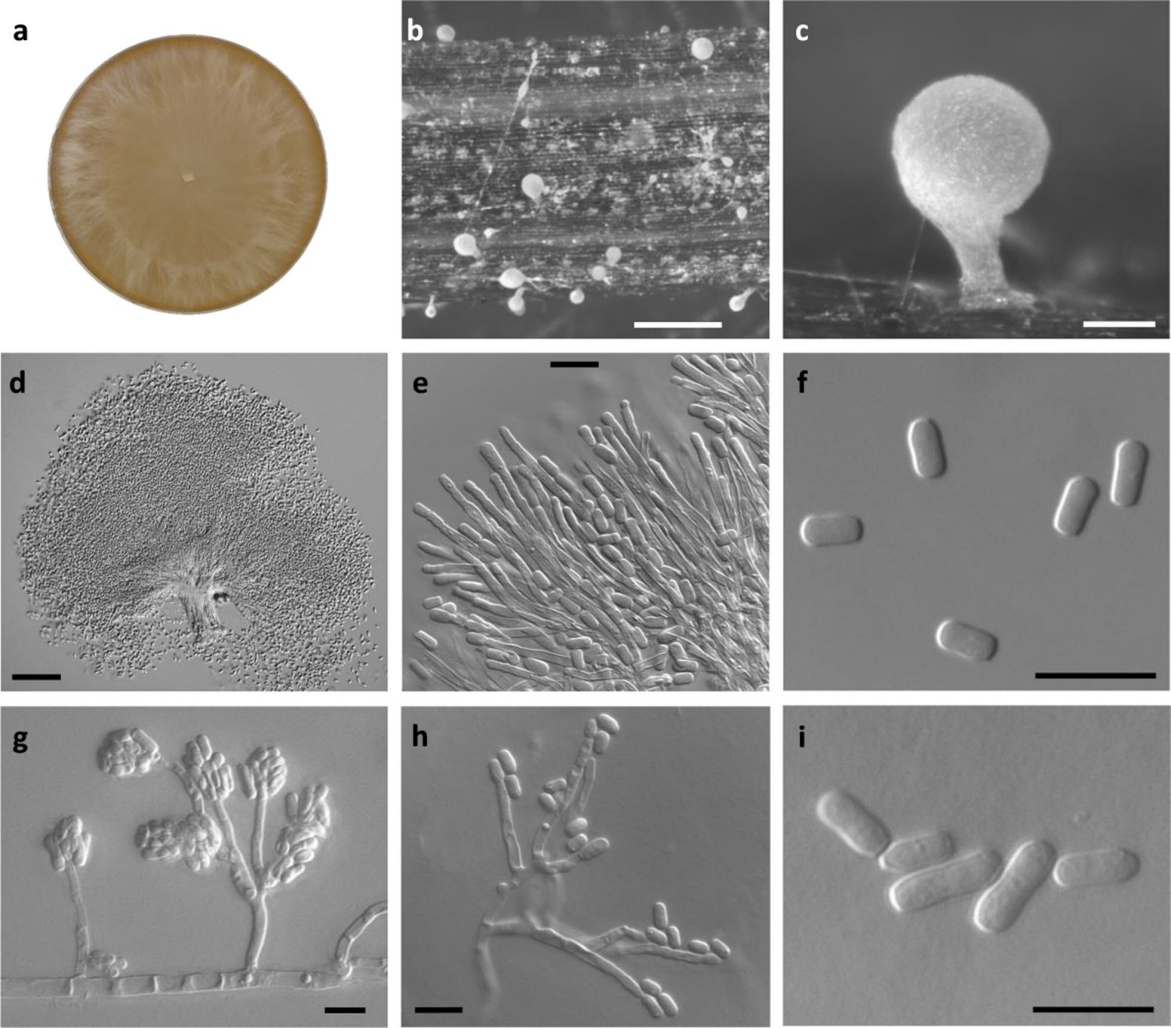
*Graphilbum ipis-grandicollis* (VPRI43762). **a** Fourteen-d culture on MEA. **b**–**d** Pesotum-like macronematal asexual morph formed on pine needle mounted in WA. **e**–**f** Conidiogenous cells (**e**) and conidia (**f**) of Pesotum-like macronematal asexual morph. **g.** Hyalorhinocladiella-like asexual morph. **h**, **i** Conidiogenous cells (**h**) and conidia (**i**) of Hyalorhinocladiella-like asexual morph. Bars: **b** = 500 µm; **c**, **d** = 50 µm; **e**–**i** = 10 µm

*Etymology: ipis-grandicollis* (Latin), referring to *Ips grandicollis*, the bark beetle vector of this species.

*Diagnosis: Graphilbum ipis-grandicollis* is phylogenetically distinct from all morphologically similar species, from which it can be readily distinguished using molecular sequence data for the ITS, beta-tubulin, elongation factor 1-alpha, and calmodulin regions (Fig. 4, Additional file 4: Fig. S1).

*Type:***Australia:***New South Wales*: Moss Vale, Belanglo State Forest (Compartment 119), from *Ips grandicollis* gallery on *Pinus radiata*, 21 Aug. 2019, *A. J. Carnegie* (Holotype VPRI43762, stored in a metabolically inactive state; ex-holotype VPRI43762).

*Description: Sexual morph* not observed. *Asexual morphs* observed both synnematous and mononematous morphs. *Synnematous morph*: pesotum-like, macronematous, hyaline or pale yellow, erect, clavate, often singular, sometimes in groups, (102–)128–213(–263) µm long including *conidiogenous* apparatus, (14–)20–38(–45) µm wide at the base; *conidiogenous cells* (17–)19–26(–31) long; *conidia* hyaline, single-celled, smooth, cylindrical to oblong, (3–)4–6(–8) × (2–)2–3(–3) µm. *Mononematous morphs*: hyalorhinocladiella-like, arising directly from mycelium; *conidiophores*, simple to strongly branched, hyaline, (27–)45–136(–170) µm long; *conidiogenous cells* (5–)13–27(–36) long; *conidia* hyaline, single-celled, smooth, oblong, often tapering at truncated base, (4–)4–8(–13) × (2–)2–3(–4) µm.

*Culture characteristics: Colonies* hyaline, circular with smooth growing edge on MEA. Mycelia submerged or flat on MEA and WA, with colonies reaching approx. 90 mm diam after 14, and 21 d, respectively. Sporulation evident after one week of growth on pine needle amended WA. Initial formation of hyalorhinocladiella-like morphs submerged in agar, with sparse formation on the agar surface. This is followed by formation of the pesotum-like morphs, first forming on the pine needle between 7 and 14 d, and eventually observed sparsely on the agar surface after 4–6 wk. Aerial hyphae bearing conidiophores, mycelial balls, and white to yellow synnemata-like clusters were also randomly observed on the two media.

*Ecology:* Isolated from beetles and beetle galleries found on various *Pinus* hosts. Host trees: *Pinus radiata*, *P. elliottii* and *P. caribaea* x *elliottii* hybrid (Additional file 2: Table S2). Insect vector*: Ips grandicollis.*

*Distribution:* Currently known only from New South Wales, Australia.

*Notes: Graphilbum ipis-grandicollis* forms part of an expanding species complex in *Graphilbum,* which includes *G. crescericum*, *G. furuicola*, *G. interstitiale*, *G. kesiyae*, and *G. sexdentatum*. Using morphology alone makes distinction between these closely related species difficult, as they share considerable similarities in the size and shapes of conidia, conidiogenous apparatus, and the asexual morphs recorded (Jankowiak et al. 2020).

*Additional specimens examined:***Australia:***New South Wales*: Moss Vale, Belanglo State Forest (Compartment 123), from *I. grandicollis* gallery on *P. radiata*, 21 Aug. 2019, *A. J. Carnegie* (VPRI43761 – culture); Inverell, Copeton Dam, from *Ips grandicollis* gallery on *P. radiata,* 25 Jul. 2019, *A. J. Carnegie* (VPRI43759 – culture); Tumut, Buccleuch State Forest (Compartment 1129), from *I. grandicollis* gallery on *P. radiata,* 2 Jun. 2019, *D. Sargeant* (VPRI43760 – culture).

## Discussion

This study was undertaken to review and update the status of ophiostomatoid fungi associated with pine and pine bark beetles in plantations in south-eastern Australia. This was achieved by reviewing reference isolates available from historic collections lodged in Australian collections, as well as including a total of 120 new isolates collected through routine forest health surveillance during the 2019–20 period. Multi-locus phylogenetic analysis using whole genome sequencing of 46 representative isolates revealed a greater than expected diversity of ophiostomatoid fungi, including 14 species from six genera in *Ophiostomatales* and a single species residing in . While most species reported in this study were already known, our study includes seven first reports and three verifications for Australia, including the identification of three previously undescribed lineages, viz. *Graphilbum ipis-grandicollis* sp. nov. (Taxon 3), *Ceratocystiopsis* sp. (Taxon 1) and a *Graphium* sp. (Taxon 15). Draft genomes of representative isolates for each taxon are also provided here to contribute to a curated reference database of ophiostomatoid fungi for Australian biosecurity.

Of the five ophiostomatoid genera previously recorded from pine in Australia, isolates were available for *Ophiostoma, Graphilbum, Leptographium s. lat.,* and *Sporothrix*. Results of the collection database searches allowed for the inclusion of reference isolates of *G. fragrans* (Taxon 2)*, Gro. huntii* (Taxon 5)*, O. angusticollis* (Taxon 7)*, O. ips* (Taxon 9), and several initially identified as *Sporothrix* species (Carnegie and Nahrung 2019; Carnegie et al. 2019). *Sporothrix* isolates obtained from the Australian reference collections were putatively identified as *S. cf. abietina* or *O. nigrocarpum* based on BLAST results of the ITS during routine diagnostics (Carnegie et al. 2019). However, our results revealed that these isolates were all a single species, identified here as *S. pseudoabietina*. Although historical records also included the morphological identification of taxa belonging to *Ceratocystiopsis* (Stone and Simpson 1987, 1990), no reference material was available of this genus. Similarly, with the recent detection of *O. pallidulum* (Carnegie and Nahrung 2019; Carnegie et al. 2019), no isolates were readily available for inclusion in this study.

Detections made during the current survey included seven taxa not previously recorded in Australia. Four were identified as known species: specifically, *Gro. radiaticola* (Taxon 6)*, O. fasciatum* (Taxon 8)*, R. deltoideospora* (Taxon 11), and *S. euskadiensis* (Taxon 12). Taxon 4 and 13 are tentatively identified here as *G*. *cf. rectangulosporium* and *S. cf. nigrograna*, respectively. Both these taxa require further taxonomic revision due to a lack of available reference data in each case. The remaining first records included the detection of the novel species *Graphilbum ipis-grandicollis* sp. nov., as well as an undescribed lineage in *Ceratocystiopsis* and *Graphium,* respectively. For both*,* our record here serves as a first verification of presence made using molecular data. These new detections were considered in a biosecurity context, following guidelines in the Emergency Plant Pest Response Deed (Plant Health Australia EPPRD 2020; https://www.planthealthaustralia.com.au/wp-content/uploads/2020/09/EPPRD-2-September-2020.pdf), and determined not to be significant pathogens nor feasible to eradicate.

Isolates of *Ceratocystiopsis* (Taxon 1) from this study grouped closely to the *Cop. ranaculosa-brevicomis* complex, with morphological and molecular sequence data suggesting that it is most closely related to a previously undescribed North American taxon, *Ceratocystiopsis* sp. 1 (Kim et al. 2005a, b; Lee et al. 2006; Plattner et al. 2009). As mentioned, previous reports of *Ceratocystiopsis* in Australia were based on morphology alone and included the putative identification of two taxa—one of which was recorded as *Cop. minuta* (Stone and Simpson 1987). A preceding study by the same authors, however, only referred to the Australian isolates in this taxon as *Ceratocystiopsis* sp. (Stone and Simpson 1990). Interestingly, the morphological descriptions made by those authors correlate with the morphological description of a *Ceratocystiopsis* sp. 1 identified in North America, which were described as *Cop. minuta-*like (Plattner et al. 2009). It is possible therefore to speculate that the isolates collected in this study represent this same taxon reported by Stone and Simpson. Most of the *Ceratocystiopsis* isolates collected in the current survey were isolated from *I. grandicollis* beetles collected from *P. ponderosa, P. caribaea* x *elliottii* and *P. taeda* in northern NSW, the same region that Stone and Simpson collected from.

The genus *Graphilbum* has recently been expanded to include 20 formally described species, which are characterised by synnematous pesotum-like and/or mononematous hyalorhinocladiella-like asexual morphs (Seifert et al. 2013; Jankowiak et al. 2020). Of the three *Graphilbum* taxa from Australian pine plantations, one was identified as *G. fragrans* (Taxon 2)*. G. fragrans* can be considered the most common species of the genus and is known to have a global distribution, including reports from Europe, Asia, North and South America, as well as Australasia (Harrington et al. 2001; Thwaites et al. 2005; Seifert et al. 2013; Chang et al. 2017; Jankowiak et al. 2020). Previous studies have suggested that *G. fragrans* comprises potentially cryptic species, based on *Graphilbum* isolates collected from New Zealand and Australia, which show differences in morphological comparisons and sequence analysis of the ITS (Harrington et al. 2001; Thwaites et al. 2005). The *G. fragrans* isolates collected in the present study shared high sequence similarity with the type strain CBS279.54 from Sweden and are clearly distinguishable from the single sequence available for the putative taxon reported as *G. aff. fragrans* from Australasia in 2005 (Harrington et al. 2001; Thwaites et al. 2005; De Beer and Wingfield 2013).

*Graphilbum ipis-grandicollis* sp. nov. (Taxon 3) grouped with several species residing in an evidently expanding complex of bark beetle associates isolated from Europe and China (Chang et al. 2017; Jankowiak et al. 2020). Multi-locus phylogenetic analysis suggests Taxon 3 is most closely related to a clade comprised of *G. crescericum*, *G. furuicola*, *G. interstitiale*, *G. kesiyae*, and *G. sexdentatum.* While ITS sequence data suggests a close relationship to a previously undescribed *Graphilbum* isolate from North America (GU129997; Fig. 4.) further investigations are required to postulate as to the true origin of this novel taxon. The species within this complex are mainly distinguishable using molecular sequence data, with only minor morphological differences observed in characteristic features such as conidia or the production of mononematous conidiophores observed for only a couple of species (Jankowiak et al. 2020). Isolates of the *Graphilbum* sp. (Taxon 4) shared an identical ITS sequence with *Graphilbum* isolates previously reported as *G. cf. rectangulosporium* in the USA (Kim et al. 2011). The US isolates were described as sterile and shown to share high levels of sequence similarity with the type strain of *G. rectangulosporium* from Japan (AB242825; Ohtaka et al. 2006; Kim et al. 2011). Cultures of the isolates in the current study did not produce either sexual or asexual characters, an observation that further validates the association with the US isolates. The lack of morphologically distinguishing characteristics in culture, as well as limited availability of alternative barcoding loci currently restricts further taxonomic placement, and so we refer to this taxon as *G. cf. rectangulosporium*.

In *Leptographium s.lat.,* isolates of *Grosmannia huntii* (Taxon 5) and *Gro. radiaticola* (Taxon 6) were collected in this study. *Gro. huntii* was first reported in Australia in 1998, when it was believed to have been introduced along with its insect vector, *H. ater* (Jacobs et al. 1998). Until now, the known distribution within Australia included Victoria and NSW in association with *H. ater* and *Hy. ligniperda*. The isolations made in the current study expand the known distribution to include Tasmania, where it was isolated from stumps in recently harvested pine plantations infested by the root-feeding bark beetle, *Hy. ligniperda*. Taxon 6 included three isolates of *Gro. radiaticola* which were collected from *P. radiata* samples infested with *H. ater* in South Australia, and *Hy. ligniperda* in Tasmania. These are the first records of *Gro. radiaticola* for Australia. *Gro. radiaticola* forms part of the *Gro. galeiformis* species complex, and has been previously reported across Eurasia (Kim et al. 2005a, b; Linnakoski et al. 2012; Jankowiak and Bilański 2013a, b; Chang et al. 2017) and throughout the Southern Hemisphere, including South America, South Africa, and New Zealand (Zhou et al. 2001, 2006; Thwaites et al. 2013; de Errasti et al. 2018).

Of the four species in our study residing within *Ophiostoma s. lat.*, only *O. ips* (Taxon 9) grouped in a well-recognised species complex. The remaining three species, *O*. *angusticollis* (Taxon 7), *O. fasciatum* (Taxon 8) and *O. pallidulum* (Taxon 10), currently group within smaller lineages that sit peripherally to *Ophiostoma s. str.* and are commonly referred to as ‘Group A’ (Chang et al. 2017; Wang et al. 2020). Species residing in Group A are consistently recorded in low numbers and known to be highly phoretic (non-permanent interaction for the purpose of travel) in their association with insects (Chang et al. 2017). *Ophiostoma fasciatum* was first described in Canada in 1972 from *Pseudotsuga menziesii* (as *Ceratocystis fasciata*; Olchowecki and Reid 1974) and *P*. *banksiana* (as *Ceratocystis spinifera*; Olchowecki and Reid 1974). The single isolate of *O. fasciatum* (Taxon 8) collected in this survey came from *I. grandicollis* collected from a *P. caribaea* x *elliottii* hybrid in northern NSW and has not been previously reported in Australia*. O. pallidulum* and *O. angusticollis* species were recently detected in NSW in 2016 and 2017, respectively (Carnegie and Nahrung 2019). The single isolate of *O. pallidulum* from our current survey was collected from a *Hy*. *ligniperda* beetle sampled in Tasmania and serves as a first report outside of NSW.

The single isolate of *Raffaelea deltoideospora* (Taxon 11) was isolated directly from galleries of *I. grandicollis* found on a *P. caribaea x elliottii* hybrid from northern NSW. *R. deltoideospora* was originally described from isolates collected from the wood of several pine species in Canada (Olchowecki and Reid 1974). Later records have found this species associated with cerambycid pupal chambers in the USA and China (Wingfield 1987; Wang et al. 2018). *R. deltoideospora* has also been reported from *P. pinaster* in the Iberian Peninsula (Villarreal et al. 2005). This is a first report for this fungus in Australia.

Results of our study revealed three species of *Sporothrix* present in Australian pine plantations. Two species belonged to the *S. gossypina* species complex, namely, *S. pseudoabietina* (Taxon 14) and *S. euskadiensis* (Taxon 12). Species within this complex are commonly isolated from bark beetle and mite associates (De Beer et al. 2016). Taxon 14 showed close association to several undescribed lineages previously referred to as either *S. cf. abietina* or *Sporothrix* sp., which included isolates from the USA, Mexico, South Africa, Poland and China (Zhou et al. 2004a; Min et al. 2009; Romón et al. 2014; Jankowiak et al. 2018). In 2019, this taxon was formally described as *S. pseudoabietina,* with the type specimen originating in China (Wang et al. 2019). Our results confirm that *S. pseudoabietina* is a commonly isolated fungus from Australian-grown pine which was first detected in 2019 (Carnegie et al. 2019). The second species belonging to this complex was identified as *S. euskadiensis,* associated with *I. grandicollis. S. euskadiensis* was first described from *P. radiata* in Spain, where isolates were associated with *Hylurgops palliatus* and *Hylastes attenuatus* (Romón et al. 2014).

The third species of *Sporothrix* identified during this study, Taxon 13, sits within species complex ‘G’ (De Beer et al. 2016). Molecular analysis and taxonomic placement for this isolate exemplifies some of the major challenges for diagnostics of ophiostomatoid fungi. Our single strain shared an identical ITS sequence with the type specimens of both *S. nigrograna* and *S. zhejiangensis* (De Beer et al. 2016; Wang et al. 2018). LSU sequences for our strain also shared high sequence similarity to those available for both *S. nebularis* and *S. nigrograna* (De Beer et al. 2016). The lack of available sequence data for other molecular regions of *S. nigrograna* limits further comparison to this species, and therefore analysis of the BT region was only possible for sequences from *S. nebularis* and *S. zhejiangensis*. Using BT alone would delimit our strain as *S. zhejiangensis*. Morphologically these species can only be distinguished by the presence or absence of a sheath on ascospores (Masuya et al. 2003; Wang et al. 2019). Until appropriate taxonomic comparison is possible, we refer to this isolate as *S*. *cf. nigrograna* due to its initial placement with *S. nigrograna* and its distinction from *S. nebularis*.

A single isolate (Taxon 15) residing within *Graphium* (Microascales) was collected from an *I. grandicollis* gallery originating from *P. elliottii* in NSW. ML analysis of our strain revealed a potentially distinct lineage that is closely related to *Gra. basitruncatum* and *Gra. carbonarium*. *Gra. basitruncatum* was first described from soil in the Solomon Islands (Okada et al. 2000), while *Gra. carbonarium* was isolated from *Pissodes* beetles on *Salix babylonica* in Yunnan, China (Paciura et al. 2010). With only this single isolate obtained we refer to this taxon as *Graphium* sp. until more isolates can be collected and studied.

Although the relationship of ophiostomatoid fungi and arthropod vectors has been extensively studied, the precise role of each taxonomic group within these systems and the specificity of these associations, are yet to be clearly defined (Chang et al. 2017; Wingfield et al. 2017b). All taxa isolated in the current study form part of species complexes and/or groupings that are consistently associated with bark beetles or other insect vectors (De Beer et al. 2016; Wingfield et al. 2017b). While our goal was to assess the diversity of ophiostomatoid fungi associated with pine bark beetles and beetle galleries collected during routine forest health surveillance, *I. grandicollis* was the more commonly encountered beetle species during the current surveillance period. This was a somewhat expected observation as, historically, *I. grandicollis* is more commonly caught in NSW than either *H. ater* or *Hy. ligniperda* (Stone et al. 2010). While this could explain the dominance observed for some of the ophiostomatoid species isolated, such as *Ophiostoma ips* and *Sporothrix pseudoabietina*, our results highlight the potential phoresy of these associations with *I. grandicollis* being linked to seven of the taxa recovered during this study (Additional file 2: Table S2)*.* While we were able to make a few general observations regarding the patterns of isolations, a more in-depth systematic review would be required for an improved understanding and description of these fungus-vector associations across Australia. More targeted surveys, particularly studies focused on the insect vectors present, are likely to reveal an even greater diversity, for example, the isolates of *Gro. radiaticola* and *Gro. huntii* were only recovered from samples that came from *H. ater* and *Hy. ligniperda* infestations.

Genome assemblies for the isolates chosen as representatives for each taxon collected in this study resulted in the addition of 12 draft genomes to the *Ophiostomatales*, and the release of the first draft genome publicly available for an isolate residing in the *Graphiaceae* (*Microascales*). The genome assembly statistics of the *Ophiostomatales* isolates collected during this study mirror those available for species residing in *Ophiostoma, Sporothrix, Graphilbum, Leptographium s.l., Ceratocystiopsis* and *Raffaelea* (DiGuistini et al. 2011; Forgetta et al. 2013; Haridas et al. 2013; Teixeira et al. 2014; van der Nest et al. 2014; Wingfield et al. 2015a, b, 2016, 2017a, 2018; D'Alessandro et al. 2016; Huang et al. 2016; Shang et al. 2016; Jeon et al. 2017; Vanderpool et al. 2018; Liu et al. 2019). Comparisons of genome statistics, specifically estimated size, GC content and number of predicted genes, generally correlate with the taxonomic placement of each species (Additional file 3: Table S3). This is evident, for example, when comparing the genomes of *Gro. galeiformis* and *Gro. radiaticola*, or *S. euskadiensis* and *S. pseudoabietina.* In both cases the size, GC content and number of predicted open reading frames (ORFs) vary marginally. There are however slight deviations evident within some genera. For example, in *Ceratocystiopsis* the genome sizes range from 20.45 to 21.30 Mb, and the number of predicted ORFs are somewhat lower for *Cop. brevicomis* (6884 ORFs) and *Ceratocystiopsis* sp. VPRI43766 (Taxon 1; 6967 ORFs) when compared to that of *Cop. minuta* (7786 ORFs).

In the modern era, fungal taxonomy relies more heavily on an integrative approach where genealogical concordance is combined with morphological examination to recognise and delimit species (Lücking et al. 2020). For taxonomists and diagnosticians looking to delineate taxa of ophiostomatoid fungi, this could include analysing anything from two to ten different gene regions (De Beer and Wingfield 2013; De Beer et al. 2014, 2016) all while comparing morphological characters that can prove extremely difficult to distinguish (Jankowiak et al. 2020). Another major challenge that was exemplified several times in this study is the inconsistency of recovering sequence data for specific taxa. As the cost of sequencing continues to decrease, the feasibility for future taxonomic surveys to include whole genome sequences should become more readily attainable. As shown in this study, future taxonomic surveys could strive to include whole genome sequence data published alongside their identifications and/or descriptions of novel taxa. Currently, in the *Ophiostomatales* the number of available genomes encompasses 38 species across 11 genera. Expanding these genomic resources provides a fundamental platform on which diagnostic and biosecurity capacity can be developed.

## Conclusions

The results of this study have uncovered a higher than expected diversity for ophiostomatoid fungi associated with pine and pine bark beetles in south eastern Australia. The current status of ophiostomatoid fungi in Australian pine plantations confirmed using molecular data has been expanded from 7 previously confirmed taxa to now include 15 verified species across six genera in the *Ophiostomatales*, as well as a single taxon identified in the *Graphiaceae* (*Microascales*). As demonstrated several times in this study, a major challenge for accurate fungal diagnostics and species delimitation is the availability of multi-locus sequence data for reference specimens. With the ever-decreasing costs of sequencing, as well as the need for multi-locus sequence data, our study provides an early example of WGS replacing standard PCR-based approaches. Future taxonomic studies could begin to look in earnest at the opportunities of providing the full complement of DNA sequence data along with the results of a given taxonomic survey. This would ensure that taxonomic studies continue to improve upon the availability of molecular data while rapidly expanding on the numbers of sampled taxa. Results of the current survey, coupled with other recent detections in Australia, illustrates the need for continued surveillance of ophiostomatoid fungi. This not only provides an important platform for recognising the underlying diversity of these fungi but allows for the establishment of an improved ophiostomatoid-specific database which will continue to develop the diagnostic capabilities for Australian biosecurity.

## Supplementary Information

**Additional file 1. Table S1.** Sampling information.

**Additional file 2. Table S2.** Host association and isolation frequencies of ophiostomatoid fungi obtained during this study.

**Additional file 3. Table S3.** Extended genome summaries of ophiostomatoid fungi which correspond with all genera obtained during current study.

**Additional file 4. Figure S1**. ML phylogeny of the CAL region for isolates residing in *Graphilbum*. Sequences generated in this study are printed in bold type with reference collection isolates coloured purple. Bold branches indicate posterior probability values ≥0.9. ML bootstrap values of ≥ 70% are recorded at nodes. T = ex-type isolates.

**Additional file 5. Figure S2.** ML phylogeny of ITS region for representative species of *Leptographium*, *Raffaelea* and *Hawksworthiomyces*. Sequences generated in this study are printed in bold type. Bold branches indicate posterior probability values ≥0.9. ML bootstrap values of ≥ 70% are recorded at nodes. T = ex-type isolates.

**Additional file 6. Figure S3.** ML phylogeny of CAL for isolates residing in *Sporothrix*. Sequences generated in this study are printed in bold type. Bold branches indicate posterior probability values ≥0.9. ML bootstrap values of ≥ 70% are recorded at nodes. T = ex-type isolates.

## Data Availability

All data generated or analysed during this study is included in this published article [and its supplementary information files] and/or is available from the corresponding author upon reasonable request.

## Abbreviations

BI: Bayesian inference
BT: β-Tubulin
CAL: Calmodulin
DAR: The NSW Plant Pathology and Mycology Herbarium
ITS: The internal transcribed spacer
LSU: The large ribosomal subunit (28S)
MEA: Malt extract agar
ML: Maximum likelihood
NCBI: National Center for Biotechnology Information
NSW: New South Wales
*s.lat.*: Sensu lato
*s.str.*: Sensu stricto
TEF: Translation elongation factor 1-α
VPRI: The Victorian Plant Pathology Herbarium
WA: Water agar
WGS: Whole genome sequencing
