# Correction to: Ophiostomatoid fungi associated with pine bark beetles and infested pines in south-eastern Australia, including *Graphilbum ipis‑grandicollis* sp. nov

**DOI:** 10.1186/s43008-021-00080-0

**Published:** 2021-09-21

**Authors:** Conrad Trollip, Angus J. Carnegie, Quang Dinh, Jatinder Kaur, David Smith, Ross Mann, Brendan Rodoni, Jacqueline Edwards

**Affiliations:** 1 School of Applied Systems Biology, La Trobe University, Bundoora, VIC 3083 Australia; 2 Department of Jobs, Precincts and Regions, Agriculture Victoria Research, AgriBio Centre, Bundoora, VIC 3083 Australia; 3 Forest Science, NSW Department of Primary Industries – Forestry, Parramatta, NSW 2150 Australia; 4 Department of Jobs, Precincts and Regions, Biosecurity and Agricultural Services, Agriculture Victoria, Cranbourne, VIC 3977 Australia

## Correction to: IMA Fungus (2021) 12:24 https://doi.org/10.1186/s43008-021-00076-w

Following the publication of the original article (Trollip et al. 2021), we were notified that there was a missing column at the end of the first section of Table 3, which included the genome information for one of the genomes generated in this study (Ophiostoma angusticollis – Genome Accession JADHKL000000000).

- Originally published table:

**Table:** **Table 3** Genome summary statistics of representative ophiostomatoid isolates sequenced in this study

Species	*Ceratocystiopsis*	*Graphilbum*			*Leptographium s. l*	
	*Ceratocystiopsis* sp.	*G. fragrans*	*G. ipis-grandicollis* sp. nov	*G. cf. rectangulosporium*	*Gro. huntii*	*Gro. radiaticola*
Taxon	Taxon 1	Taxon 2	Taxon 3	Taxon 4	Taxon 5	Taxon 6
Sequenced strain	VPRI43766	VPRI43528	VPRI43762	VPRI43763	VPRI43530	VPRI43523
GenBank Accession	JADHKF010000000	JADHKG010000000	JADHKH010000000	JADHKI010000000	JADHKJ010000000	JADHKK010000000
Total reads after QC	27,063,242	50,135,289	49,429,518	46,778,484	94,294,776	114,599,394
Number of scaffolds	79	237	178	117	254	85
Longest contig (Mb)	1.54	999,956	1,555,217	1,343,814	1,099,032	2,583,828
Est. genome size (Mb)	20.45	34.04	24.02	23.61	28.05	27.56
N50 (bp)	471,680	323,198	601,306	298,270	343,842	883,760
L50	12	32	13	22	25	11
# N's per 100 kbp	10	7	14	6	8	5
GC (%)	61.48	55.75	55.53	60.91	54.63	57.09
Avg. coverage depth	197	218	305	296	493	619
No. of predicted genes	6967	9034	7221	7270	7836	7867
Est. gene density	341	265	301	308	279	286
Complete BUSCO (%)	95.20	97.10	95.60	95.70	97.40	96.40
Complete BUSCO (n)	3636	3706	3647	3651	3716	3680
Complete—single	3631	3699	3644	3648	3710	3673
Complete—duplicated	5	7	3	3	6	7
Fragmented	17	23	29	18	17	21
Missing	164	88	141	148	84	116

Species	*Ophiostoma s. l*			*Raffaelea*	*Sporothrix*			*Graphium*
	*O. fasciatum*	*O. ips*	*O. pallidulum*	*R. deltoideospora*	*S. euskadiensis*	*S. cf. nigrograna*	*S. pseudoabietina*	*Graphium *sp.
Taxon	Taxon 8	Taxon 9	Taxon 10	Taxon 11	Taxon 12	Taxon 13	Taxon 14	Taxon 15
Sequenced strain	VPRI43845	VPRI43529	VPRI43846	VPRI43720	VPRI43754	VPRI43755	VPRI43531	VPRI43844
GenBank Accession	JADHKM010000000	JADHKN010000000	JADHKO010000000	JADHKP010000000	JADHKQ010000000	JADHKR010000000	JADHKS010000000	JADHKT010000000
Total reads after QC	51,287,508	37,261,588	36,666,452	35,927,882	29,627,312	37,781,794	36,719,768	28,160,580
Number of scaffolds	36	208	473	120	92	125	51	490
Longest contig (Mb)	2,360,849	1,117,630	543,815	1,965,135	1,881,930	1,307,617	3,412,636	1,008,604
Est. genome size (Mb)	22.54	26.01	32.66	30.95	36.13	27.34	35.20	31.65
N50 (bp)	966,108	283,044	126,120	416,364	790,753	398,989	1,285,428	190,968
L50	9	29	84	25	16	23	9	49
# N's per 100 kbp	4	8	12	18	11	12	8	12
GC (%)	65.26	56.88	57.93	54.55	52.70	59.54	53.27	50.12
Avg. coverage depth	341	209	167	173	122	205	154	132
No. of predicted genes	7498	7470	8757	7428	9348	7908	9190	9482
Est. gene density	333	287	268	240	259	289	261	300
Complete BUSCO (%)	96.60	96.90	97.20	94.90	98.10	97.20	97.80	96.60
Complete BUSCO (n)	3688	3696	3710	3621	3743	3710	3734	3686
Complete—single	3685	3690	3705	3613	3739	3703	3729	3679
Complete—duplicated	3	6	5	8	4	7	5	7
Fragmented	16	16	28	30	10	23	12	40
Missing	113	105	79	166	64	84	71	91
- Corrected table:

**Table:** **Table 3** Genome summary statistics of representative ophiostomatoid isolates sequenced in this study

Species	*Ceratocystiopsis*	*Graphilbum*			*Leptographium s. l*		*Ophiostoma s. l*
	*Ceratocystiopsis* sp.	*G. fragrans*	*G. ipis-grandicollis* sp. nov	*G. cf. rectangulosporium*	*Gro. huntii*	*Gro. radiaticola*	*O. angusticollis*
Taxon	Taxon 1	Taxon 2	Taxon 3	Taxon 4	Taxon 5	Taxon 6	Taxon 7
Sequenced strain	VPRI43766	VPRI43528	VPRI43762	VPRI43763	VPRI43530	VPRI43523	VPRI43764
GenBank Accession	JADHKF010000000	JADHKG010000000	JADHKH010000000	JADHKI010000000	JADHKJ010000000	JADHKK010000000	JADHKL010000000
Total reads after QC	27,063,242	50,135,289	49,429,518	46,778,484	94,294,776	114,599,394	10,454,088
Number of scaffolds	79	237	178	117	254	85	317
Longest contig (Mb)	1,540,000	999,956	1,555,217	1,343,814	1,099,032	2,583,828	651,020
Est. genome size (Mb)	20.45	34.04	24.02	23.61	28.05	27.56	23.80
N50 (bp)	471,680	323,198	601,306	298,270	343,842	883,760	236,362
L50	12	32	13	22	25	11	34
# N's per 100 kbp	10	7	14	6	8	5	14
GC (%)	61.48	55.75	55.53	60.91	54.63	57.09	57.89
Avg. coverage depth	197	218	305	296	493	619	61
No. of predicted genes	6967	9034	7221	7270	7836	7867	8022
Est. gene density	341	265	301	308	279	286	337
Complete BUSCO (%)	95.20	97.10	95.60	95.70	97.40	96.40	96.60
Complete BUSCO (n)	3636	3706	3647	3651	3716	3680	3686
Complete—single	3631	3699	3644	3648	3710	3673	3680
Complete—duplicated	5	7	3	3	6	7	6
Fragmented	17	23	29	18	17	21	18
Missing	164	88	141	148	84	116	113

Species	*Ophiostoma s. l*			*Raffaelea*	*Sporothrix*			*Graphium*
	*O. fasciatum*	*O. ips*	*O. pallidulum*	*R. deltoideospora*	*S. euskadiensis*	*S. cf. nigrograna*	*S. pseudoabietina*	*Graphium sp.*
Taxon	Taxon 8	Taxon 9	Taxon 10	Taxon 11	Taxon 12	Taxon 13	Taxon 14	Taxon 15
Sequenced strain	VPRI43845	VPRI43529	VPRI43846	VPRI43720	VPRI43754	VPRI43755	VPRI43531	VPRI43844
GenBank Accession	JADHKM010000000	JADHKN010000000	JADHKO010000000	JADHKP010000000	JADHKQ010000000	JADHKR010000000	JADHKS010000000	JADHKT010000000
Total reads after QC	51,287,508	37,261,588	36,666,452	35,927,882	29,627,312	37,781,794	36,719,768	28,160,580
Number of scaffolds	36	208	473	120	92	125	51	490
Longest contig (Mb)	2,360,849	1,117,630	543,815	1,965,135	1,881,930	1,307,617	3,412,636	1,008,604
Est. genome size (Mb)	22.54	26.01	32.66	30.95	36.13	27.34	35.20	31.65
N50 (bp)	966,108	283,044	126,120	416,364	790,753	398,989	1,285,428	190,968
L50	9	29	84	25	16	23	9	49
# N's per 100 kbp	4	8	12	18	11	12	8	12
GC (%)	65.26	56.88	57.93	54.55	52.70	59.54	53.27	50.12
Avg. coverage depth	341	209	167	173	122	205	154	132
No. of predicted genes	7498	7470	8757	7428	9348	7908	9190	9482
Est. gene density	333	287	268	240	259	289	261	300
Complete BUSCO (%)	96.60	96.90	97.20	94.90	98.10	97.20	97.80	96.60
Complete BUSCO (n)	3688	3696	3710	3621	3743	3710	3734	3686
Complete—single	3685	3690	3705	3613	3739	3703	3729	3679
Complete—duplicated	3	6	5	8	4	7	5	7
Fragmented	16	16	28	30	10	23	12	40
Missing	113	105	79	166	64	84	71	91

The original article has been corrected.
