# Supplementary figures and images for: Ophiostomatoid fungi associated with pine bark beetles and infested pines in south-eastern Australia, including Graphilbum ipis-grandicollis sp. nov
Source: IMA Fungus. 2021 Sep 1;12:24. doi: 10.1186/s43008-021-00076-w (PMC8408996; doi:10.1186/s43008-021-00076-w)

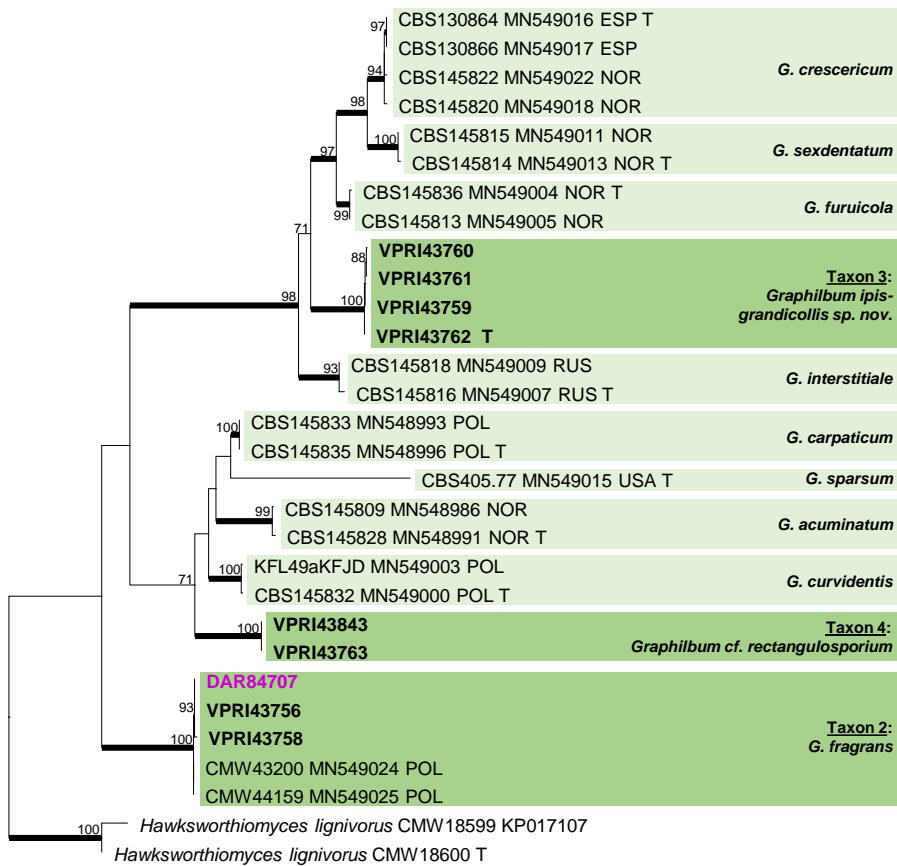

Supplement: Supplementary file 4 — Additional file 4. Figure S1. ML phylogeny of the CAL region for isolates residing in Graphilbum. Sequences generated in this study are printed in bold type with reference collection isolates coloured purple. Bold branches indicate posterior probability values ≥0.9. ML bootstrap values of ≥ 70% are recorded at nodes. T = ex-type isolates. [file 43008_2021_76_MOESM4_ESM.pdf]

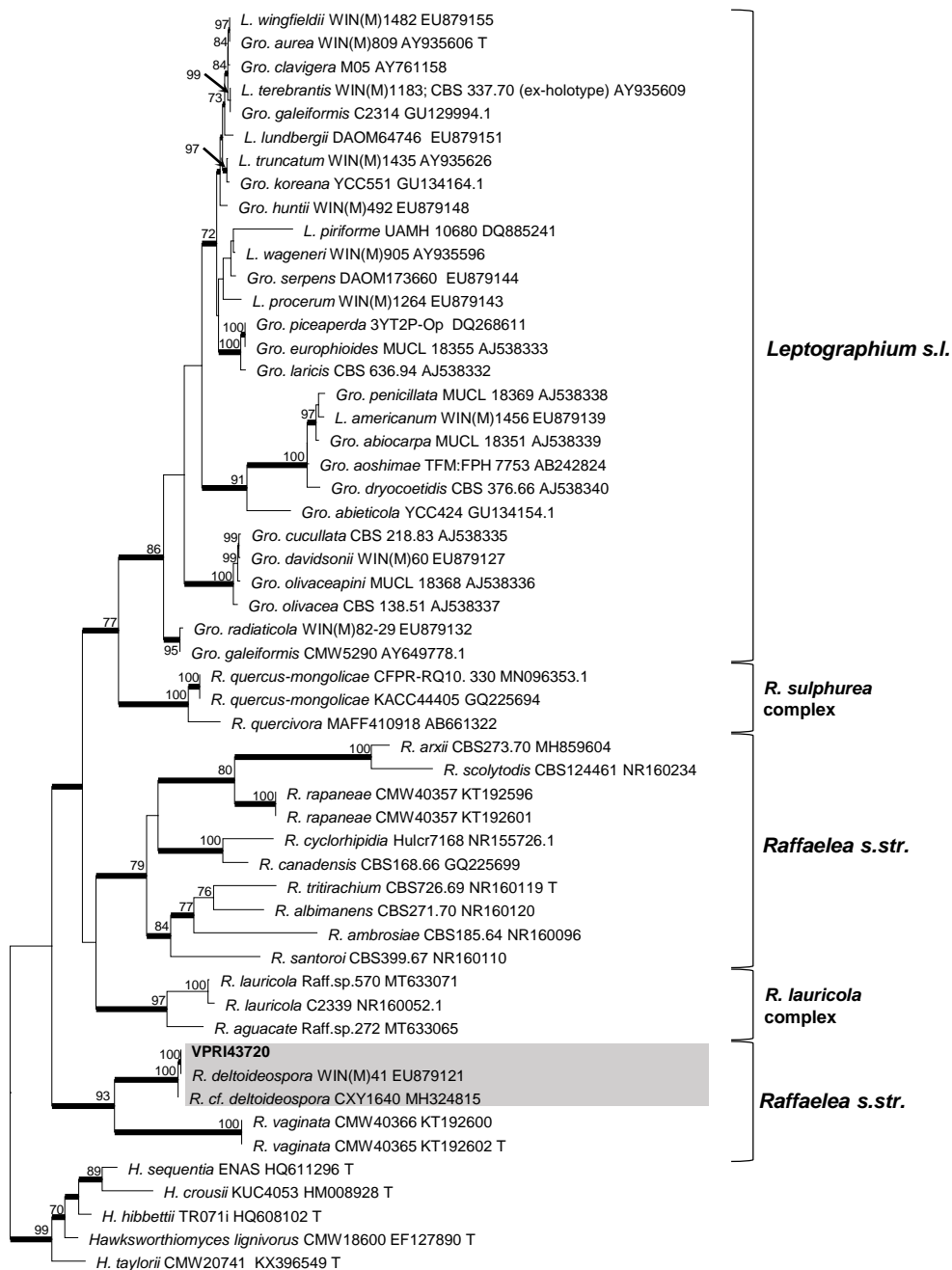

Supplement: Supplementary file 5 — Additional file 5. Figure S2. ML phylogeny of ITS region for representative species of Leptographium, Raffaelea and Hawksworthiomyces. Sequences generated in this study are printed in bold type. Bold branches indicate posterior probability values ≥0.9. ML bootstrap values of ≥ 70% are recorded at nodes. T = ex-type isolates. [file 43008_2021_76_MOESM5_ESM.pdf]

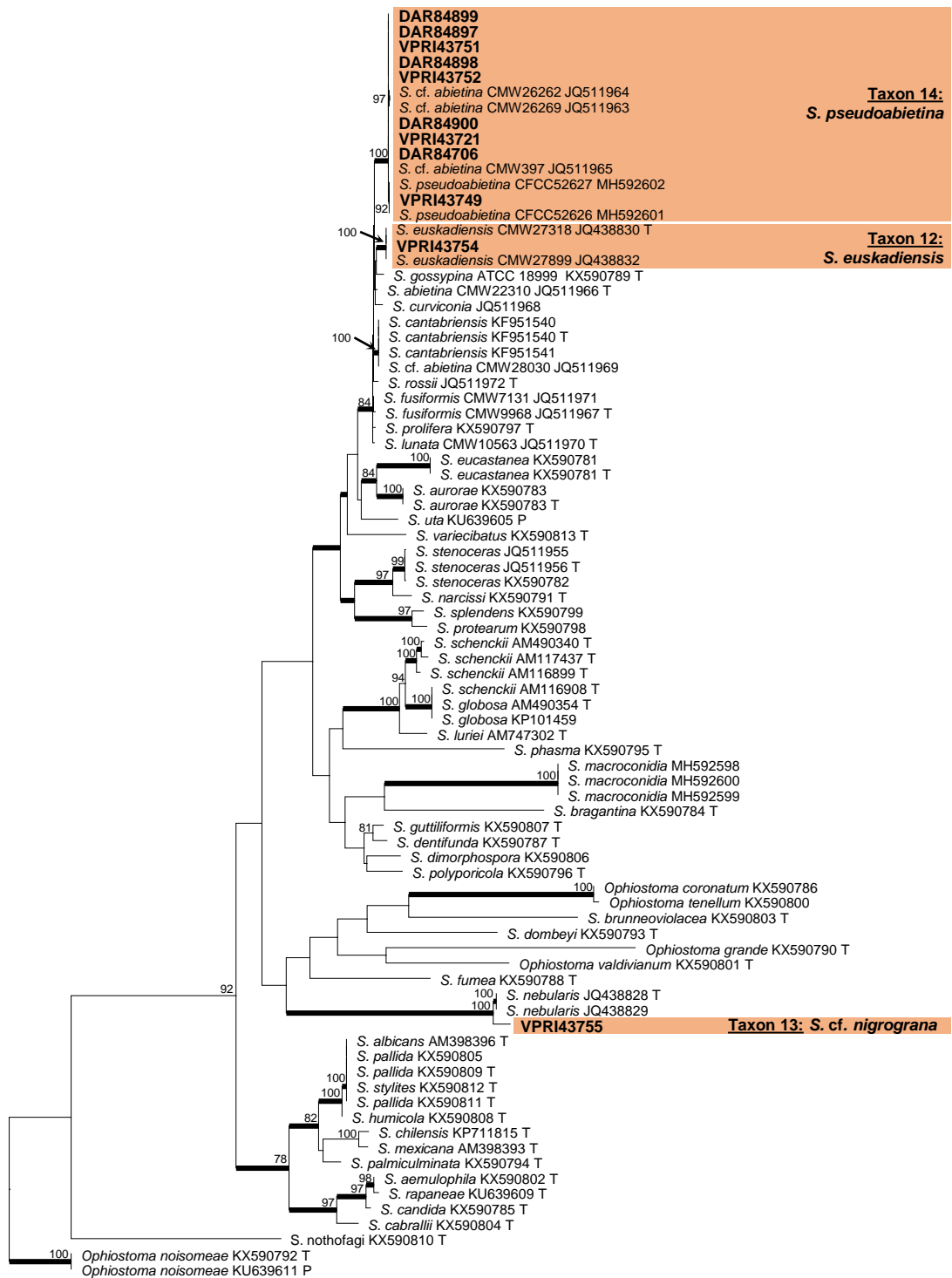

0.3

Supplement: Supplementary file 6 — Additional file 6. Figure S3. ML phylogeny of CAL for isolates residing in Sporothrix. Sequences generated in this study are printed in bold type. Bold branches indicate posterior probability values ≥0.9. ML bootstrap values of ≥ 70% are recorded at nodes. T = ex-type isolates. [file 43008_2021_76_MOESM6_ESM.pdf]
